# 2025 Update to the Female Athlete Triad Coalition Consensus Statement Part 2: Clinical Guidelines for Screening, Diagnosis, Treatment, and Return to Play for Adolescents and Adults

**DOI:** 10.1007/s40279-025-02332-0

**Published:** 2025-12-31

**Authors:** Nancy I. Williams, Mary Jane De Souza, Madhusmita Misra, Aurelia Nattiv, Elizabeth Joy, Michelle Barrack, Emily A. Ricker, Sasha Gorrell, Kristen J. Koltun, Emma O’Donnell, Rebecca J. Mallinson, Ana Carla C. Salamunes, Kary Woodruff, Michael Fredericson, Franziska Plessow

**Affiliations:** 1 Women’s Health and Exercise Laboratory, Noll Laboratory, Department of Kinesiology, Pennsylvania State University, University Park, PA 16802 USA; 2 Misra Laboratory, Division of Pediatric Endocrinology, Department of Pediatrics, University of Virginia, Charlottesville, VA USA; 3 Division of Sports Medicine, Departments of Family Medicine and Orthopaedic Surgery, University of California Los Angeles, Los Angeles, CA USA; 4 Lore Health, Salt Lake City, UT USA; 5 Department of Family and Consumer Sciences, California State University, Long Beach, Long Beach, CA USA; 6 Consortium for Health and Military Performance, Department of Military and Emergency Medicine, F. Edward Hébert School of Medicine, Uniformed Services University, Bethesda, MD USA; 7 Henry M. Jackson Foundation for the Advancement of Military Medicine, Inc., Bethesda, MD USA; 8 Department of Psychiatry and Behavioral Sciences, Weill Institute for Neurosciences, University of California San Francisco, San Francisco, CA USA; 9 Neuromuscular Research Laboratory, Department of Sports Medicine and Nutrition, University of Pittsburgh, Pittsburgh, PA 15203 USA; 10 School of Sport and Exercise Health Sciences, National Centre of Sports and Exercise Medicine, Loughborough University, Loughborough, Leicestershire UK; 11 Pennsylvania State University Harrisburg, Harrisburg, PA USA; 12 Nutrition and Integrative Physiology, University of Utah, Salt Lake City, UT USA; 13 Division of Physical Medicine and Rehabilitation, Department of Orthopaedic Surgery, Stanford University School of Medicine, Stanford, CA USA; 14 Stanford Prevention Research Center, Stanford University, Stanford, CA USA

## Abstract

This is the second of two publications comprising the 2025 update to the 2014 Consensus Statement on treatment and return to play guidelines on the Female Athlete Triad (Triad). This paper pairs with the *2025 Update to the Female Athlete Triad Coalition Consensus Statement Part 1: State of the Science and Introduction of a New Adolescent Model* (Sports Medicine, 2025), to focus on evidence-based revisions for screening, diagnosis, treatment, and clearance and return to play. Revised recommendations for managing eating disorders (ED)/disordered eating (DE) and non-pharmacological and pharmacological treatment of bone loss and abnormal menstrual cycles are included, as are the most recent clearance and return to play recommendations, inclusive of adolescent athletes. Recent research supports the adoption of revised criteria for defining and treating energy deficiency, moving away from the concept of an energy-availability threshold. Energy deficiency-induced menstrual disturbances can be reversed with a moderate increase in food intake and modest weight gain, but restoration of menses alone is not associated with high rates of ovulation or increased ovarian steroid levels until multiple consecutive normal length menstrual cycles are achieved. Revised guidelines for the diagnosis and treatment of functional hypothalamic oligo/amenorrhea are included with guidance on the confounding effects of hyperandrogenemia. Gynecological age and psychological stress are factors impacting the individual susceptibility to the Triad. The bone health spectrum of the Triad now includes bone stress injuries. Routes of administration via epidermal patch versus oral for pharmacological treatment of low bone density are discussed. The diagnosis, treatment, and return to play approaches for adolescents with the Triad are unique compared with those employed for adults and require age-appropriate clinical guidelines. The strength of the evidence-based statements is graded using an accepted taxonomy in which randomized controlled trials and observational data are considered the highest level of evidence.

## Key Points

**Table:** 

Bone stress injuries can result from chronic energy deficiency and or hypoestrogenemia associated with exercise-associated menstrual disturbances and are now included in the Female Athlete Triad (Triad) model.
Randomized trials demonstrate that the rates and physiological mechanisms of induction versus reversal of Triad conditions differ and, accordingly, monitoring and treatment approaches should be specific relative to induction or reversal and specific for each Triad spectrum.
Assessments of energy deficiency are critical to identifying Triad risk, and use of the revised terminology not tied to the concept of an energy availability threshold is recommended to categorize the severity/risk of energy deficiency.
The diagnosis, treatment, and return to play approaches for adolescents with the Triad are unique compared to those employed for adults and require age-appropriate clinical guidelines.
Translational tools such as the Preparticipation Physical Evaluation, validated eating behavior questionnaires, the Triad clearance and return to play algorithm, and if resources allow, laboratory measurements of energy deficiency, should be incorporated into sports medicine teams’ approaches to Triad prevention and management.

## Introduction

This Consensus Statement, *2025 Update to the Female Athlete Triad Coalition Consensus Statement Part 2: Clinical Guidelines for Screening, Diagnosis, Treatment and Return to Play for Adolescents and Adults*, is the second article in a two-part series updating the 2014 Consensus Statement on treatment and return to play of the Female Athlete Triad from the Female and Male Athlete Triad Coalition [2–4]. This 2025 update represents a consensus of recommendations for physicians, sports medicine practitioners, and other healthcare providers. Two publications comprise this update. The first paper, *2025 Update to the Female Athlete Triad Coalition Consensus Statement Part I: State of the Scienc****e**** and Introduction of a New Adolescent Model* [1]*,* provides a detailed update on the scientific underpinnings of the Female Athlete Triad, introduces revisions to the current Triad model, and introduces a new Triad model specific to the adolescent female athlete. This second paper provides updated information on diagnosis and clinical practice, treatment guidelines, and a revised clearance and return to play cumulative risk factor assessment for adolescent and adult female athletes. Both 2025 papers represent a consensus of the state of the science and recommendations for physicians, sports medicine practitioners, other healthcare providers and researchers that was developed by the Female and Male Athlete Triad Coalition.

### The Consensus Process and Evidence Grading

An independent expert panel of 16 individuals with demonstrated expertise in the various topics related to Triad science and previous members of Triad consensus papers was convened to consolidate evidence-based data and develop recommendations. We drew on emerging data and the expertise of the diverse writing team of clinicians, sports dietitians, and scientists. The expertise of the group deemed necessary to properly address the current state of knowledge of Triad science included experts in the following subject areas: adolescent and adult eating disorders, psychology of eating behavior, biological and cognitive psychology, exercise physiology, cardiovascular physiology, kinesiology, nutrition, women’s health, pediatric and adult endocrinology, bone health, orthopedics, physical therapy, nutrition, reproductive medicine, and clinical sports medicine. No funding was involved in the recruitment or work of the panel. No conflicts of interest were declared.

Consensus was established using a strategy similar to that utilized by the 2014 Female Athlete Triad Coalition Consensus Statement [4]. The consensus process included one in-person group meeting, several virtual video meetings, and e-mail communications. The topics discussed in the Consensus Statement were divided amongst the writing team, and a leader was established for each section. Section leaders then worked with their teams to draft each section and develop evidence-based statements. Section leaders presented their team’s literature review findings and a group discussion followed to develop a final consensus on the evidence-based statements. Each evidence-based statement underwent extensive discussion during video conferences. Exact language was developed, edited, and finalized. Grading of each statement was discussed and agreed upon only after a thorough group discussion. Next, online confidential voting on all evidence-based statements was used to determine consensus. Responses included “agree,” “neutral,” or “disagree.” If > 85% of writers “agreed” on a given evidence statement and there were no instances of “disagree,” no further discussion of the statement ensued. Discussion occurred if ≤ 85% of writers “agreed” and there were instances of “neutral” or “disagree.” Discussion resulted in either a revision of the statement or a deletion of the statement. Voting on revised statements was repeated with subsequent discussion and revision of statements, until all questions achieved > 85% of writers responding with “agree” with no instances of “disagree.” If recommendations from peer reviewers of the manuscript included revision of any evidence-based statements, the process of voting for those statements was repeated. The strength of the evidence-based statements was graded using a taxonomy in which randomized controlled trials (RCTs) and observational data are considered the highest level of evidence, as utilized in the American College of Sports Medicine position stands and by the Agency for Healthcare Research and Quality [5, 6]. The specific evidence scoring criteria were:

- Evidence Level A: Consistent pattern of findings based on substantial data from RCT(s) and/or observational studies.
- Evidence Level B: Strong evidence from RCT and/or observational studies, but with some inconsistent results from the overall conclusion.
- Evidence Level C: Evidence from a smaller number of observational and/or uncontrolled or nonrandomized trials which is generally suggestive of an overall conclusion.
- Evidence Level D: Insufficient evidence for categories A–C; panel consensus judgment.

The co-chairpersons (MJD and NIW) of the Consensus Statement organized notetaking from each consensus meeting and compiled manuscript drafts. Drafts of the document were then circulated to the entire writing team for review and further editing ensued following each video conference until a final document was agreed upon after achieving consensus by the entire writing team. The entire paper was reviewed by independent reviewers as per journal guidelines for Sports Medicine.

### What is the Value of a Consensus Statement Focused Specifically on the Female Athlete Triad?

Since the 2014 Female Athlete Triad Consensus Statement publication [3, 4], advances in scientific and clinical understandings of the Triad have progressed, necessitating an updated Consensus Statement summarizing the state of the science and recommended clinical practice. While no theoretical model of a medical condition is perfect, Triad researchers have always strived to present a model that is not only scientifically accurate but useful for both clinicians and practitioners. Attention to the Triad is also warranted given the consistent increase in the number of girls and women participating in sport. For example, as of 2022, there has been a record number of female student-athletes i.e., 230,518 competing in the US National Collegiate Athletic Association (NCAA) [7]. Moreover, the prevalence of eating disorders (EDs) and disordered eating (DE) behaviors, i.e., primary drivers of the Triad, has risen sharply in the general population since the COVID-19 pandemic, particularly among college-aged young adults [8, 9]. Additionally, a recent study found that athletes, when compared to the general population, are less likely to seek treatment for EDs due to stigma, accessibility, and sport-specific barriers [10]. Attention to the issue has grown as high-profile athletes have spoken out regarding their Triad experiences on social media [11].

Lastly, the Triad is the foundation of the broadly described condition known as Relative Energy Deficiency in Sport (REDs). REDs was described in 2014 as a condition of impaired physiological and/or psychological functioning involving numerous physiological and performance outcomes experienced by female and male athletes and caused by exposure to low energy availability (EA) [12–14]. Initially, REDs was framed as a more all-encompassing model than the Triad, inclusive of many more physiological endpoints that were purported to be causally tied to low EA in both females and males. While the REDs model includes the three components of the Triad, i.e., low EA with or without EDs/DE, menstrual function, and bone health, a concern is that the REDs model also includes several outcomes for which no RCTs are available to support their causal link to low EA, for example, gastrointestinal and immune outcomes, and the model is applied to men despite the absence of the availability of data to describe causal links to low EA in men [15, 16]. Moreover, the clinical relevance of the Triad is obscured in the REDs model because the manner in which it is depicted minimizes its relevance to simply a minor component of the REDs model. However, in the 2023 REDs update, Triad conditions are actually the major presentation of REDs described as being caused by “problematic low EA” [12]. Specifically, the “primary indicators” of a REDs diagnosis rendering the highest risk categorization, i.e., associated with “problematic EA” are menstrual disturbances or low testosterone, low bone mineral density (BMD) or bone stress injuries (BSIs), and EDs. That said, the recently published REDs update has since eliminated any mention of the Female or Male Athlete Triad and does not include clinically significant information specifically focused on Triad prevention, diagnosis, treatment, and return to play [12]. Despite this, the components of the Triad, i.e.,  reproductive disturbances, and BMD/BSI still represent REDs outcomes with the highest prevalence and the largest body of evidence as to the known clinical outcomes with an established causal link to energy deficiency. Thus, a diagnosis of REDs at its most severe presentation continues to be based on diagnosing the presence of the Triad, as its components are the “most well-documented sequelae of problematic low EA”[12]. While research addressing the REDs model is ongoing, the sustained relevance of its foundational origin, i.e., the Female and Male Athlete Triad, has been recognized by others [15–19]. Furthermore, a recent publication has formally questioned the validity of the scientific evidence supporting the REDs model [16]. The latter publication describes the lack of supporting empirical scientific evidence and other concerns that are associated with the REDs model, i.e., the low quality of evidence supporting the association between low EA and many of the REDs outcomes and the likelihood that other factors, such as sleep, stress, and overtraining, may contribute significantly and independently to these outcomes. In summary, the sustained relevance and clinical importance of the Triad, when considered in the context of the less well scientifically defined concept known as REDs, underscore the importance of this updated Triad Consensus Statement which continues to evolve based on advances in the supporting science.

### Presentation of the 2025 Update of the Adult Female Athlete Triad Model

**Fig. 1:**
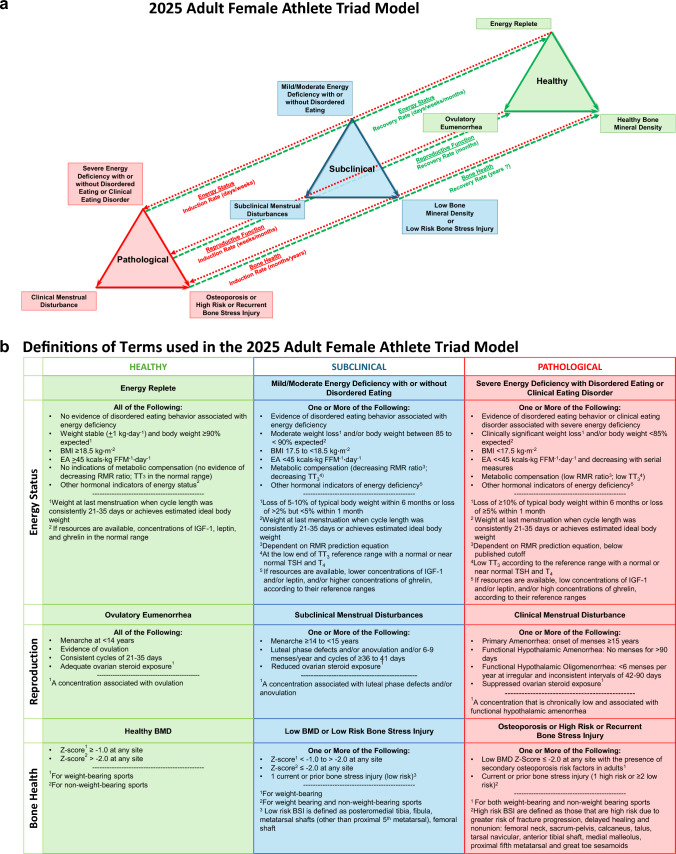
**a** The updated 2025 Adult Female Athlete Triad Model. The three inter-related components of the Female Athlete Triad are energy status, reproductive function and bone health. The Female Athlete Triad is initiated by exposure to varying degrees of energy deficiency with or without disordered eating/eating disorders with primary pathological outcomes to the reproductive and skeletal systems. Separate bi-directional arrows indicate the different rates of induction and recovery of each pathological outcome. The lack of data supporting the potential for complete recovery of bone health is noted by “?”. Chronic hypoestrogenemia resulting from energy deficiency induced reproductive suppression independently affects bone health. Updates include revised terminology describing energy status, i.e., energy deficiency instead of energy availability. Bone stress injury is now included in the bone health spectrum. *See Figure **b** for an explanation of the detailed features of the Triad Continuum Components. **b** Definitions of terms used in the updated 2025 Adult Female Athlete Triad Model. Detailed descriptions of the terminology used to describe the healthy, subclinical, and pathological states of each Triad component have been revised according to recent research. *BMD* bone mineral density, *BSI* bone stress injury, *BMI* body mass index, *EA* energy availability, *IGF-1* insulin-like growth factor-1, *RMR* resting metabolic rate, *TSH* thyroid stimulating hormone, *TT*_*3*_ total triiodothyronine, *T*_*4*_ thyroxine

As the expert panel considered the state of the science as of 2025, several updates to the Triad continuum model (Fig. 1a, b) were identified, as described below.

#### Energy Deficiency and “Metabolic Compensation” are added to the Energy Status Continuum to describe Adaptations that Reflect Energy Conservation

There is no validated or universally accepted approach to describe or quantify the body’s overall energy status or the specific partitioning of oxidative fuels to various physiological systems such as growth, reproduction, locomotion, or thermoregulation. As such, for the purposes of this Consensus Statement, we are defining terms relating to the energy continuum in the context of the scientific literature to date pertaining to the Triad where the specific focus is on understanding the metabolic status most aligned with the induction and reversal of Triad conditions. That said, advances in research support revised terminology describing the concepts and quantitative metrics comprising the energy status continuum. Notably, the term “energy deficiency” is now used in association with the energy status spectrum instead of “low EA.” Low EA (defined as the difference between energy intake and exercise energy expenditure normalized for fat free mass (FFM)) is now one of several indicators of energy deficiency in addition to low body weight and/or weight loss, low body mass index (BMI), and/or indications of metabolic compensation. The rationale for this change is that EA is difficult to reliably estimate and can fluctuate widely from day to day [20]. Moreover, the calculation of EA alone is insufficient to accurately describe energetic status, as it does not capture body energy stores or physiological indications of metabolic compensation, a new term introduced in this revised Consensus Statement.

“Metabolic compensation” refers to factors that reflect adaptive processes to reallocate the body’s energy resources for the purpose of energy conservation. Indications of metabolic compensation are observed in response to the loss of body weight that may or may not represent a new chronically low body weight set-point [21, 22] or low BMI [23, 24]. Metabolic compensation also includes the suppression of resting metabolic rate (RMR) normalized for FFM [22], a reduced ratio of measured to predicted metabolic rate (mRMR/pRMR ratio), and/or sustained evidence of decreases in concentrations of metabolic hormones, or levels that are low or at the lower end of the reference range, such as total triiodothyronine (TT_3_), insulin-like growth-factor-1 (IGF-1), leptin [25–27], and increased ghrelin [28]. It is important to note that these hormonal and metabolic adaptations, described as metabolic compensation, are often associated with a state of energy balance, underscoring that observations of weight stability and or efforts limited only to assessments of energy balance can be misleading. As well, body weight stability should not be interpreted as evidence of energy balance, as plasma volume expansion with training [29] and increased body water stored with training induced increases in glycogen storage [30] can contribute to body weight increases and as such may mask mild energy deficiency. Compensatory metabolic adaptations can occur over days to weeks in energy-deficient individuals and appear to be reversible, although less is known regarding the exact timeframes over which specific metabolic parameters return to levels associated with being energy replete [4, 22, 26, 27, 31].

#### Bone Stress Injuries are added to the Bone Health Continuum

The addition of bone stress injuries (BSIs) to the model reflects well-established data demonstrating that Triad components (e.g., menstrual disturbances, low body weight, DE) are consistently associated with increased risk of BSI, relating to a spectrum of bone injury from repetitive mechanical loading that results in structural inflammation, fatigue, and local bone pain which can precipitate a frank cortical stress fracture [32–34]. Notably, stress fracture rates in functional hypothalamic oligo/amenorrhea (FHOA) exercising women exceed those of women with anorexia nervosa, a population that is typically considered at high risk for low BMD and fracture [35] [36]. As such, the subclinical range of the bone health continuum now encompasses “Low BMD or Low Risk BSI,” and the pathological range encompasses “Osteoporosis or High Risk or Recurrent BSI” as defined in Fig. 1b.

#### Ovarian Steroid Hormones, Functional Hypothalamic Oligomenorrhea (FHOA), and Bidirectional Lines Indicating Rates of Induction and Recovery are added to the Reproductive Function Continuum

The addition of ovarian steroid hormones to the reproductive continuum is warranted in light of RCT evidence and other studies demonstrating the importance of nutritional interventions for recovery of not only menses, but also the recovery of ovarian steroid concentrations and their downstream effects on bone [37, 38]. Thus, even in the face of increased menstrual frequency following amenorrhea, full recovery of ovarian steroids is not always observed [38]. As such, resumption of menses, ovarian steroid concentrations, and the presence or absence of ovulation are all of clinical significance and warrant consideration when defining reproductive recovery. Evidence from RCTs also suggests that transitions along these menstrual-related spectra occur at different rates for induction and recovery, with induction occurring faster than recovery (see Fig. 1a). The addition of functional hypothalamic oligomenorrhea to the pathological end of the continuum demonstrates that care should be taken to rule out a hybrid-type presentation of oligomenorrhea associated with biochemical hyperandrogenism, as opposed to oligomenorrhea of hypothalamic origin, similar in etiology to FHA [39, 40]. As such, we now refer to functional hypothalamic amenorrhea and oligomenorrhea as functional hypothalamic oligo/amenorrhea (FHOA).

#### Rates of Change are Depicted for the Induction and Recovery of Clinical Outcomes within the Adult Model

Since the previous Triad Consensus Statement was published, more evidence has accumulated to demonstrate the rate of induction/recovery of clinical outcomes, thus supporting refinements to the model [27, 37]. It is becoming evident that the rate of change for a particular outcome may be different if an individual is moving from “healthy” to “pathological” (induction), or from “pathological” to “healthy” (recovery). For instance, the REFUEL RCT demonstrated that within 12 months of refeeding, previously oligo/amenorrheic women can have an increased frequency of menses [37]. In most participants who recovered menses, this increased frequency was noted in the first 3 months of refeeding. However, 12 months of refeeding was not long enough to re-establish regular ovulatory menstrual cycles or normal patterns of ovarian hormone exposure [38]. Notably, after 12 months of refeeding with a high proportion of participants resuming menses (defined as the occurrence of menses in amenorrheic participants or an increase in the number of menses in oligomenorrheic participants), BMD did not improve, perhaps indicating that the time frame over which refeeding needs to occur to improve bone health exceeds 12 months [41]. As well, it is possible that the volume of increased energy intake required to impact bone is greater, or that the energetic starting point, i.e., baseline body weight or body fat, or baseline RMR, or hormonal status of TT3 or IGF-1, may impact the time course of recovery. In another study with moderate caloric restriction and exercise training over three months, the induction of menstrual disturbances in previously ovulatory young, untrained women was associated with metabolic changes such as weight loss, reductions in RMR, decreases in TT_3_, IGF-1, and leptin and increases in ghrelin. The magnitude of energy deficit was proportional to the frequency of observed menstrual disturbances, all within 3 months [27]. In the updated model figure, rates of change are noted along “Induction Rate” and “Recovery Rate” lines.

#### A Question Mark (?) Indicates the Uncertainty of Bone Mineral Density (BMD) Recovery, which Requires Clarification through a Stronger Evidence Base

It is questionable whether recovery of BMD is possible following increased energy intake. After a 12-month intervention (REFUEL RCT) of approximately 350 kcal/day in oligo/amenorrheic exercising women, BMD did not improve at any site despite increased weight and fat mass compared to the control group [41]. However, a longer duration of increased energy intake, a greater magnitude of energy intake, specificity of varying macronutrient composition, and consideration of baseline demographics may impact the potential for improvement in BMD.

#### New Details Focused on the Adolescent and an Accompanying Model Figure are Introduced

**Fig. 2:**
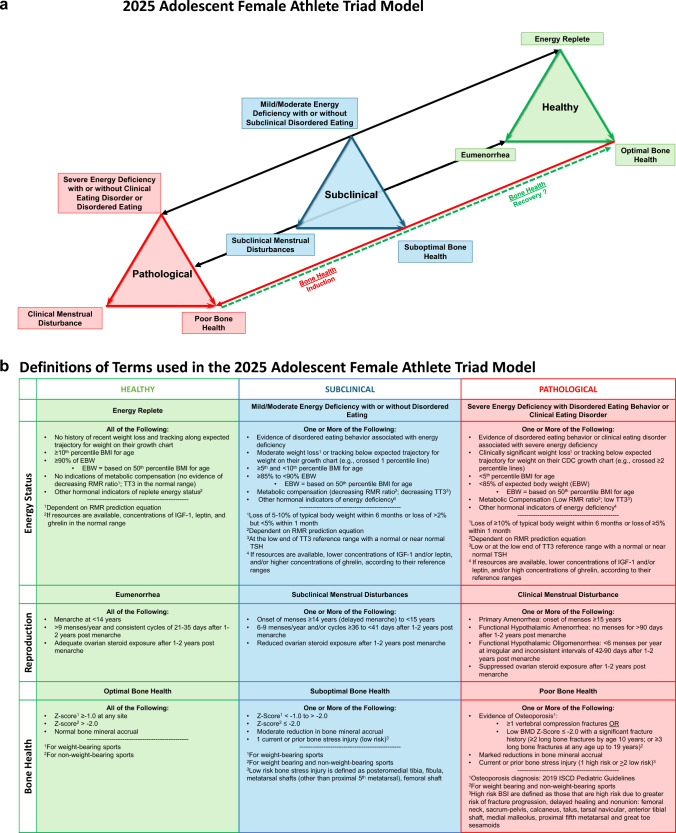
**a** The new 2025 Adolescent Female Athlete Triad Model. The three inter-related components of the Female Athlete Triad are energy status, reproductive function and bone health. The adolescent Female Athlete Triad is initiated by exposure to varying degrees of energy deficiency with or without disordered eating/eating disorders with primary pathological outcomes to the reproductive and skeletal systems. Chronic hypoestrogenemia resulting from energy deficiency induced reproductive suppression independently affects bone health. Bi-directional arrows indicate both induction and recovery of the pathological outcomes for energy status and reproductive status. Separate arrows and “?” for bone health indicate the lack of data supporting the potential for complete recovery of bone health. *See Figure **b** for an explanation of the detailed features of the Triad Continuum Components. **b** Definitions of terms used in the new 2025 Adolescent Female Athlete Triad Model. Detailed descriptions of the terminology used to describe the healthy, subclinical, and pathological states of each Triad component have been revised according to recent research. *BMD* bone mineral density, *BSI* bone stress injury, *BMI* body mass index, *EA* energy availability, *EBW* expected body weight, *IGF-1* insulin-like growth factor-1, *ISCD* International Society for Bone Densitometry, *RMR* resting metabolic rate, *TSH* thyroid stimulating hormone, *TT*_*3*_ total triiodothyronine

Female adolescent athletes may be more severely impacted by the Triad given potential disruptions to bone mineral accrual and risk of developing a low peak BMD, which underscores the need for early identification, treatment, and prevention efforts. Energy deficiency in adolescent female athletes can result in delayed menarche or primary amenorrhea, secondary amenorrhea, low BMD, reduced bone accrual, and low peak bone mass [42–48]. An adolescent Triad model is introduced in this update (Fig. 2a, b).

## Diagnostic Updates to the Energy Continuum

### General Comments and Definitions of Energy Terminology

**Fig. 3:**
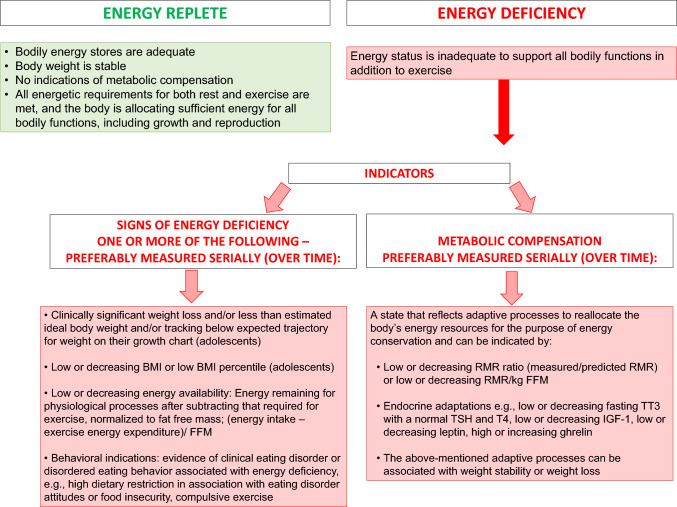
Definitions for revised terminology for the energetic components of the 2025 Adult and Adolescent Female Athlete Triad Models. *BMI* body mass index, *FFM* fate free mass, *IGF-1* insulin-like growth factor-1, *RMR* resting metabolic rate, *TSH* thyroid stimulating hormone, *TT*_*3*_ total triiodothyronine, *T*_*4*_ thyroxine

Understanding the causal role of energy status in the induction and reversal of Triad outcomes requires clear and consistent use of terminology. We have defined the following terms in the *2025 Update to the Female Athlete Triad Coalition Consensus Statement Part 1: State of the Science and Introduction of a New Adolescent Model* [1] and advocate for consistent use in all future Triad literature. The terms include energy replete, energy deficiency, EA, and metabolic compensation, i.e., measures indicating suppression of resting metabolism (Fig. 3). Key research advances since the previous Female Athlete Triad Consensus Statement include more specific recommendations for monitoring athletes’ energy status, including the use of indicators of metabolic compensation and a discussion of the limitations of assessing EA, particularly with respect to the use of an EA threshold.

### What are the Current Recommendations to Screen for and Diagnose Energy Deficiency?

As Williams et al. [49] have described, the ideal marker of energy deficiency is one that can be accurately and objectively measured and is reflective of compensatory metabolic adaptations to chronic energy deficiency. Furthermore, an ideal marker of energy deficiency should be an accurate biomarker of downstream effects on health and/or performance outcomes, such as suppression of reproductive function, bone loss, bone stress injuries, or decrements in sport performance [49]. That said, there is no perfect indicator of energy deficiency. Questionnaires aimed at identifying the risk of energy deficiency include the Low Energy Availability in Females Questionnaire (LEAF-Q), although there are limits to its validity (see Sect. 6.3.2) [50] and the more recent FED-Q questionnaire [51], which was validated against circulating concentrations of TT3. Biological indicators of energy deficiency, generally preferred over self-report questionnaires, include low or decreasing body weight, BMI, percent body fat, EA, metabolic hormone concentrations (e.g., TT3, leptin, IGF-1), and/or the RMR ratio or RMR normalized for fat free mass, and/or presence of DE behaviors, all of which must be interpreted with important caveats in mind. While single time measurements of body weight or BMI may indicate a high risk of undernutrition or chronic energy deficiency if body weight is < 85% of expected body weight, or BMI is < 17.5 kg/m^−2^ [52], or if an adolescent’s BMI for age is < fifth percentile [53], serial measurements are recommended to document downward trajectories. However, body weight and BMI may not be ideal indicators of energy status because they can remain stable even in a state of energy deficiency and when metabolism is suppressed due to other compensatory mechanisms [22]. Importantly, as described in *2025 Update to the Female Athlete Triad Coalition Consensus Statement Part 1: State of the Science and Introduction of a New Adolescent Model* [1], the use of an EA “threshold” (e.g. < 30 kcal/kg FFM/day), is not recommended due to more recent data demonstrating that menstrual disturbances occur in exercising women at EA levels above this threshold [54, 55] and due to the limitations of quantifying EA in real-life situations versus under laboratory conditions [20, 56]. Signs of metabolic compensation may include low or decreasing normalized resting metabolic rate (RMR/kg FFM or mRMR/pRMR) [22], low or low normal fasting metabolic hormone concentrations, such as total triiodothyronine (TT3), leptin, and insulin-like growth factor 1 (IGF-1) or increases in ghrelin [25, 27, 28]. Notably, energy deficiency has been shown to vary with training demands and phase of a competitive season and recommendations for intervention may be dependent on the context and/or other factors [57, 58]. Regarding the identification of energy deficiency through the presence or absence of ED/DE behaviors, it should be noted that not all pathways to energy deficiency include ED/DE [59].

***Evidence-Based Statement:*** Measures of energy deficiency are most useful when obtained serially within an individual at the same time of day. ***Grade:*** Level B.

***Evidence***-*B****ased Statement:*** Evidence of energy deficiency is best obtained by documenting serial decreases in at least one or more of the following physiological or behavioral measures: body weight, % expected body weight, BMI, percentile BMI for age (adolescents), EA, or increases in measures of eating attitudes and behaviors reflective of dietary restriction (see Sect. 3) and if resources allow, measures of metabolic compensation such as decreases in the RMR ratio, and or decreases in fasting endocrine measures such as total or free T3, IGF-1, leptin, and or increases in ghrelin. ***Grade:*** Level B.

***Evidence-Based Statement:*** If a single time point is all that is available, measurements of body weight or BMI may indicate a high risk of undernutrition or chronic energy deficiency if body weight is < 85% of expected body weight, or BMI is < 17.5 kg/m^−2^, EA is much less than 45 kcal∙kg/FFM^−1^∙day ^−1^, if an adolescent’s BMI for age is < fifth percentile, or if the RMR ratio or TT3 are low. ***Grade:*** Level B.

### If Resources Allow, what is the Best Approach to Identify Metabolic Compensation?

Evidence supporting the physiological basis for incorporating the concept and measurement of metabolic compensation as an indicator of energy deficiency is detailed in *2025 Update to the Female Athlete Triad Coalition Consensus Statement Part 1: State of the Science and Introduction of a New Adolescent Model* [1]. Metabolic compensation refers to factors that reflect adaptive processes to reallocate the body’s energy resources to conserve energy.

The initial use of the mRMR/pRMR ratio in Triad populations [60, 61] used the Harris-Benedict prediction equation [62], in which a mRMR/pRMR ratio below 0.90, indicating that the measured RMR is less than 90% of the predicted value, was associated with proxies of energy deficiency, such as menstrual disturbances [63], high drive for thinness [63, 64], cognitive restraint [65], high peptide YY [66], low TT_3_ [61], low measured EA [67], and high LEAF-Q scores [68]. However, different prediction equations [68–71] incorporate different variables into the prediction algorithm; therefore, 0.90 should not be used as a universal threshold to determine metabolic compensation [72]. The specific cutoff used for the mRMR/pRMR ratio depends on the prediction equation used to calculate predicted RMR [73]. When used appropriately, the mRMR/pRMR ratio has been shown to provide reliable data with good specificity and sensitivity in women when measured against circulating concentrations of TT_3_ over time [72, 73]. Notably, the use of laboratory measures, such as RMR or serum measures of metabolic hormones, is limited due to high cost, time, equipment, and specialized expertise. Going forward, portable, handheld devices for monitoring metabolic rate may hold promise for easier athlete monitoring if properly validated against laboratory methods.

### Assessment of Eating Attitudes and Behaviors may serve as a Proxy Indicator of Energy Status

Using various psychometric measures of eating attitudes and behaviors in lieu of laboratory-derived measurements of energy status can serve as useful proxy indicators of energy deficiency. For example, drive for thinness and dietary restraint, which when excessive, are cognitive symptoms of EDs, have demonstrated significant associations with RMR and TT_3_ assessments in exercising women [63, 74]. However, despite these associations, not all pathways to energy deficiency may include DE per se. Compulsive exercise, as “driven” physical activity that is undertaken to avoid negative affect (e.g., guilt or anxiety) [75] and/or in the interest of weight/shape control [76] should be considered a contributor to energy deficiency, especially in the context of DE/EDs [77, 78]. Inadvertent undereating is also a plausible pathway to energy deficiency, given that exercise may suppress hunger and/or food intake may be limited by athletes’ lack of access to food, their busy training schedules, or other factors [59, 79]. Time-restricted eating (e.g., intermittent fasting) and other methods to achieve purposeful weight loss to prepare for competition or based on training periodization should also be considered as a contributor to energy deficiency [80, 81]. Lastly, extended periods of energy deficiency throughout the day have been associated with signs of metabolic compensation [82, 83].

***Evidence-Based Statement:*** Psychometric measures of eating attitudes and behaviors, for example, drive for thinness and/or dietary restraint, can serve as useful proxy indicators of energy deficiency. ***Grade:*** Level B.

***Evidence-Based Statement:*** Behaviors such as compulsive exercise, inadvertent undereating, time-restricted eating, for example, intermittent fasting and/or undereating for prolonged periods during the day, are associated with energy deficiency. ***Grade:*** Level C.

## Screening, Diagnostic, and Treatment Updates for Eating Disorders (EDs) and Disordered Eating (DE)

### General Comments

The Triad can be both defined and exacerbated by DE behaviors. DE constitutes a range of irregular eating behaviors that may or may not warrant a diagnosis of a specific ED [84] and may or may not collectively meet clinical diagnosis of an ED. Notably, engagement in DE can endanger physical health both acutely [85] and chronically [84, 86] and is associated with a greater likelihood of meeting criteria for a clinical ED in the future [87, 88]. ED diagnoses described in the Diagnostic and Statistical Manual of Mental Disorders, Fifth Edition, Text Revision (DSM-5-TR) that may commonly arise within athlete samples include anorexia nervosa, bulimia nervosa, binge eating disorder, Avoidant/Restrictive Food Intake Disorder (ARFID), and Other Specified Feeding and Eating Disorder (OSFED) [89]. Regardless of weight status, these ED diagnoses include patterns of dietary restriction, potential for malnutrition, and body image concerns related to weight and shape that may motivate DE (with the exception of ARFID, where fear of weight gain is typically not present) [89].

While commonly thought to have relatively low prevalence rates compared to other serious psychiatric disorders, the lifetime prevalence rate of EDs for females < 20 years of age is ~ 13% (3.6% with a variant of anorexia nervosa) [90]. Anorexia nervosa is among the most lethal of psychiatric disorders, with a standardized all-cause mortality ratio (observed deaths/expected deaths) estimate of approximately 6 over the last few decades compared to the general population [91–93] and higher lifetime risk of suicide attempts observed among women with an ED diagnosis compared to those without [94]. EDs contribute a substantial global disease burden [95], and for a majority of patients, require protracted and costly treatment [96]. EDs can have a chronic presentation, with illness duration extending to over 20 years for over half of those afflicted [97]. That said, evidence supports the need to recognize and treat EDs in younger patients as they can have better outcomes than adults [98]. Given these extensive negative sequelae, appropriate and timely management of subclinical or clinical ED symptoms (e.g., weight restoration) can both aid in resolving the Triad and prevent lifelong negative health consequences.

There have been several key research advances relevant to eating pathology and athletes since the previous Female Athlete Triad Consensus Statement was published. Those prioritized here include: (1) improved understanding of the diagnosis of atypical anorexia nervosa, a variant of OSFED that may commonly present in certain athlete types; (2) increased awareness of the prevalence rates of DE among athletes, and individual- and system-level factors that may contribute to risk for engagement in these ED-related behaviors; and (3) an acknowledged need for athlete-specialized ED treatment, as well as timely referral, screening, and other primary prevention efforts to reduce the likelihood of DE and EDs in athlete populations.

### What are the Recommended Screening Strategies for Athletes with EDs and DE?

Sports dietitians, sports medicine physicians, eating behavior specialists, and/or mental health professionals are the appropriate professionals to evaluate the risk and/or presence of EDs and DE. Detailed recommendations for screening tools are given below. Of note, a majority of widely used ED/DE screens were developed and normed in young adult White females. As such, general trends across samples likely reflect the limitations of assessment tools that lack cultural specificity [99]. In addition to noting the importance of inclusivity and intersectionality in ED/DE assessment more broadly [99], effective ED/DE screening of athletes requires assessment tools that are validated in these populations. Denial and minimization of ED/DE symptoms are common and contribute to under-reporting [100]. As athletes may be particularly sensitive to the perceived risks of self-reporting ED/DE and even more hesitant to disclose symptoms, care should be taken to protect the privacy of the athlete, with clear communication around who will be receiving and acting upon the information they might provide. During the screening process, if systemic issues related to ED/DE risk within the athletic environment are suspected (e.g., encouragement by coaches to engage in inappropriate weight loss behaviors), the burden of addressing the issue should not be solely placed on the athletes themselves, but should include others on the sports medicine and sports science team including team physicians, athletic trainers, strength and conditioning coaches, sport dietitians, and other sport performance personnel. Training should incorporate increasing education among these key stakeholders about how to prevent and promote detection of ED/DE [101, 102].

Screening tools specifically for ED/DE in athletes may be selected based on the amount of time and allowable assessment burden. Evidence based tools include the 28-item Eating Disorders Examination Questionnaire (EDE-Q) [103] and the Brief Assessment of Stress and Eating (BASE-10) [104], which are broadly available and valid instruments for identifying ED/DE symptomology. As noted above, it may be more appropriate to use tools that are specifically designed and intended for use in athlete populations. A recent comprehensive review of new screening tools that have been used in athlete samples [105] suggests that two measures in particular demonstrate suitability: for young adults, the Eating Disorder Screen for Athletes (EDSA) [106] and for adolescents, the Disordered Eating Scale for Athletes-6 (DESA-6) [107]. These measures are relatively new, and future research is warranted. While these aforementioned instruments cannot diagnose ED/DE, they can identify athletes at risk who should be referred for further assessment in clinical interviews [79].

More comprehensive screening for Triad components, including DE, menstrual dysfunction, and bone health, can occur during athletes’ Preparticipation Physical Evaluation (PPE) [108] (Table 1). Physicians and advanced practice providers (physician assistants (PAs) and nurse practitioners (NPs)) are the appropriate professionals to administer the PPE. The PPE monograph, co-developed by several US Sports Medicine professional organizations, includes several questions that inquire about ED/DE, menstrual dysfunction and bone health [108]. The PPE can be used in conjunction with the Cumulative Risk Assessment (CRA) (discussed in Sect. 6); however, it should be noted that decisions resulting from the use of the CRA should only be made by physicians.

**Table 1:** Preparticipation physical evaluation (PPE)

Area	Questions
Eating disorders	Do you worry about your weight? Are you trying to or has anyone recommended that you gain or lose weight? Are you on a special diet or do you avoid certain types of foods or food groups? Have you ever had an eating disorder?
Menstrual dysfunction	Have you ever had a menstrual period? How old were you when you had your first menstrual period? When was your most recent menstrual period? How many periods have you had in the past 12 months?
Bone health	Have you ever had a stress fracture or an injury to a bone, muscle, ligament, joint, or tendon that caused you to miss a practice or game? Do you have a bone, muscle, ligament, or joint injury that bothers you?

The PPE is required by the NCAA, the National Federation of State High School Associations (NFHS), and most state high school athletic associations. While most states require a PPE at some point during high school sports, the timing and frequency of the PPE are variable [109].

***Evidence-Based Statement***: All female athletes should be screened for Triad risk factors. The Preparticipation Physical Evaluation (PPE), administered by healthcare providers, can effectively assess bone health, menstrual dysfunction, and ED/DE risk; other psychometric tools should be considered to assess ED/DE attitudes and behaviors, serving as a proxy for energy status. ***Grade:*** Level B.

### How Should Athletes with Suspected EDs and DE be Evaluated?

#### Physician Assessment

Athletes with an ED/DE or suspected of having an ED/DE should undergo a comprehensive assessment by a physician, including history, physical examination, and diagnostic testing (as deemed necessary). Physical examination should be conducted to identify nutrition-specific signs of energy deficit, and/or eating disorder behaviors. Notably, ED/DE may co-occur or evolve from other health conditions. Determination should be made whether the athlete can be treated in an outpatient setting, or if she requires a higher level of care due to clinical instability [79, 109]. Further assessment should be performed by a sports dietitian and mental health professional to better understand eating-related attitudes, beliefs, behaviors, body image concerns, and assess for co-morbid mental health conditions [79, 109].

#### Nutrition Evaluation

A registered dietitian, preferably a sports dietitian, should conduct a complete nutrition assessment on athletes screened at risk of ED/DE [109]. As with all dietary assessments, anthropometric, biochemical, clinical, dietary, and environmental information should be gathered. Although weight is one anthropometric measurement assessed, dietitians should avoid a weight-centric approach; an athlete may present with stable weight or have an appropriate amount of fat mass and yet may be chronically energy deficient. Additional considerations specific to athletes at risk of ED are included in Table 2. These additional factors considered individually may not reflect DE; however, all information taken together should be assessed to provide a more comprehensive picture.

**Table 2:** Additional considerations specific to athletes at risk for eating disorders (EDs)/disordered eating (DE)

Assessing the athlete’s weight history (e.g., do they have a history of significant weight fluctuations?)
Assess menstrual history and current menstrual status Assess the athlete for signs of chronic energy deficiency (see Sects. 2.2, 2.3) Collect a diet history, and evaluating the athlete’s relationship with food: Is the athlete able to consume sufficient energy and nutrients to meet their training needs? Can the athlete be flexible with dietary intake and adapt to changes based on nutrient needs and food availability? Does the athlete engage in detailed calorie and nutrient tracking? Can the athlete eat socially and without stress or anxiety? Does the athlete restrict certain foods or food groups, report multiple food intolerances, or severely restrict the time period during which they eat each day? How does the athlete view their body, and is negative body image a driver of food choices (consider standardized ED questionnaires)?

#### Mental Health Evaluation

Mental health evaluation for a suspected or confirmed ED/DE depends on certain presenting factors including age, acknowledgment of presenting problems, motivation for change, social support, severity of symptoms, and access to resources. The initial goal of the mental health evaluation is to determine the degree of imminent risk in order to make an informed decision regarding referral for appropriate treatment (if indicated). Equally important is an assessment of current psychiatric stability related to suicidal ideation, including more passive thoughts of resignation, for example, “If I should die (from current symptoms) that would be just fine with me.” Depending on the degree of imminent risk, consideration of appropriate level of care is necessary: inpatient, residential, partial hospitalization, intensive outpatient, outpatient treatment, or no treatment indicated at this time. Given the strong relationship between a history of sexual trauma and the development of an ED [110] [111], this should be addressed by a trusted member of the athlete care team in an environment that ensures privacy. From this point, mental health evaluations may vary depending on the therapeutic orientation of the evaluating provider and available resources.

Comorbid presentations that frequently co-occur with ED/DE include anxiety and mood disorders [112], obsessive–compulsive disorder [113], and post-traumatic stress disorder [114], among others. These comorbid psychiatric conditions and how ED symptoms interact with these conditions should be considered when developing a comprehensive treatment plan. A detailed description of dietary patterns, activity, and substance-use behaviors, along with self-reported body image and ED-related features, are important to assess. Gathering insight into the psychological function of the behaviors (e.g., grounding/calming, emotional numbing) and the interrelationship with other symptoms (e.g., mood, anxiety) is another goal of evaluation. A discussion of the individual’s goals for treatment can highlight potentially effective interventions and identify possible areas of treatment resistance. A patient who can acknowledge their place within the Transtheoretical Model or Stages of Change [115] may exhibit more readiness to engage in therapeutic intervention for a given presenting problem.

***Evidence-Based Statement:*** When an ED or DE is suspected, best practices should include evaluation by providers from three specialty areas: physician (medical concerns), registered dietitian, preferably a sports dietitian (nutritional concerns), and mental health professional, preferably a provider with ED expertise (mental health concerns). ***Grade:*** Level B.

### What Updated Diagnostic Information do we know about how ED/DE Presents among Athletes?

Although the Triad has been traditionally associated with anorexia nervosa, athletes may experience malnutrition and energy deficiency in the context of any type of ED, particularly given that restriction is a core symptom of all EDs, including binge eating disorder [89]. Moreover, recent research over the past decade underscores that DE behaviors, such as binge eating, self-induced vomiting, and compulsive exercise, are more common in athletes compared to non-athlete peers [10, 116]. Revisions to the 2013 edition of the DSM-5 replaced the DSM-IV Eating Disorder Not Otherwise Specified (EDNOS) diagnosis—thought to be a “catch-all” category for ED presentations that were not better accounted for—with the OSFED designation. OSFED encompasses ED presentations that are not “specified” or that are considered “subthreshold” (e.g., behavioral frequencies that might be too low to otherwise meet criteria for an ED). Research over the past decade has been increasingly focused on atypical anorexia nervosa, a diagnosis that falls under the OSFED category. For individuals with atypical anorexia nervosa, all criteria for anorexia nervosa are met, except that despite significant weight loss, the individual is still within a normal weight range for their age, sex, and height [89]. The diagnosis might be a misnomer, as recent systematic reviews conclude that both lifetime and point prevalence rates of atypical anorexia nervosa appear to be as high or higher than anorexia nervosa [117] and that mean scores on standardized interviews or self-report measures of ED psychopathology were higher for those diagnosed with atypical anorexia compared to anorexia nervosa [118].

***Evidence-Based Statement***: Individuals with OSFED, including atypical anorexia nervosa, may not present at a low weight, relative to population-based norms, but may experience very serious medical and psychological consequences secondary to malnutrition. ***Grade:*** Level A.

***Evidence-Based Statement***: Athletes can be diagnosed with all types of ED, present with any weight, and exhibit any of the behavioral features of recognized ED diagnoses. ***Grade:*** Level A.

### What are the Treatment Options for Athletes with ED/DE?

There are many well-established barriers to engaging with mental health services among athletes (e.g., athletes’ busy schedules, stigma, availability of providers, cost of care) [119], many of which are exacerbated when considering ED treatment specifically [120]. Compounding these challenges for athletes is a common lack of ED/DE literacy among athletes and coaches [121], an errant belief that engaging in DE and/or low weight will enhance performance [122], greater mental health stigma compared to non-athletes [123], and fear that treatment seeking will demonstrate weakness [124] and/or jeopardize their ability to participate in certain competitive events.

In recognition of these barriers, growing calls from those who study and care for athletes acknowledge that athletes are a unique population with unique mental health needs (e.g., [125, 126]). Some recent interventions have been developed to reduce ED/DE among athletes [127–129]; however, research devoted to the *treatment* of EDs among athletes is still limited [130, 131], with only a few interventions having been implemented specifically among athletes to date [132].

Global international guidelines recommend Family-based Treatment (FBT) as the first line intervention for youth with EDs [133, 134]. For those for whom FBT is ineffective or contraindicated (e.g., caregivers unable to participate in treatment), Cognitive-Behavioral Therapy, Enhanced for EDs (CBT-E) is also recommended. Although evidence-based treatments that target the core biology of anorexia nervosa do not yet exist [135], CBT-E has received considerable empirical support for transdiagnostic EDs [136], with growing support for the use of psychotherapy approaches like Dialectical Behavior Therapy and Acceptance and Commitment Therapy [137, 138]. However, even recommended evidence-based approaches leave roughly 50% of youth (the most common period of ED onset) unremitted at the end of a standard course of treatment [139]. While these numbers reflect treatment response in the general population, it is quite possible that athletes may experience poorer treatment response to standard treatments, due to their unique needs and presentation. With respect to pharmacological treatment of EDs, there are US Food and Drug Administration (FDA)-approved pharmacotherapies for bulimia nervosa (fluoxetine) and binge-eating disorder (lisdexamphetamine) [140], but data on the systematic evaluation of these approaches in athletes is lacking.

Considering the currently available treatment options, recent qualitative research and commentary provide a rationale for athlete-specific treatment programs that can address athlete-specific risk factors that impact onset and maintenance of symptoms and treatment response (identified above), as well as risk factors that occur within the athletic environment (e.g., [121, 131, 141]). Some ED treatment centers in the USA now also feature an athlete track, and there are a limited number of programs that are tailored specifically for athlete ED care [131, 142, 143].

***Evidence-Based Statement***: Treatment for ED among athletes should be designed to minimize acknowledged barriers to entry and address athlete and sport-specific factors that contribute to engagement in DE, and treatment responses. ***Grade:*** Level B.

### What Treatment Standards are Appropriate for Athletes with ED and DE?

Multidisciplinary care is the gold standard for patients with ED/DE, including a physician, registered dietitian, and mental health professional [79, 109]. Coordinated care should be carried out by professionals with training and experience in ED care, and when working with athletes, knowledge of the demands and risks of sport participation as it relates to ED/DE behaviors. Table 3 outlines the roles and responsibilities of each member of the athlete care team.

**Table 3:** Roles and responsibilities of the athlete care team

Role	Responsibilities
Physician (ideally sports medicine physician)	Assess physical and mental health Prescribe medication Coordinate care with athlete care team Clearance and return to play decision making
Registered Dietitian (ideally sport dietitian)	Assess dietary intake Determine energy and nutrient needs Assess eating attitudes and behaviors Develop and provide meal plan
Mental Health Professional	Assess mental health Provide psychotherapy Assess eating attitudes, behaviors, and other potential ED symptoms, e.g., self-induced vomiting; exercise driven by weight/shape concerns Provide evidence-based treatment for EDs (i.e., Family-based Treatment; Cognitive Behavioral Therapy—enhanced for EDs)
Athletic Trainer	Assess athlete status day-to-day as directed by a physician Assist in alternative training (if indicated) Assist in return to sport
Physical Therapist	Prescribe and monitor rehabilitation from bone stress injury Assess and treat muscle imbalances, issues with gait mechanics

*ED* eating disorder

Resumption of activity following treatment for a clinical ED should be scaffolded and include the perspective of all members of the treatment team who can assess both physical and psychological readiness. Return to play guidelines should be developed collaboratively with the athlete and carefully monitored. The athlete should begin retraining in a graded fashion, with participation that is contingent on a willingness to continue to adhere to the nutritional plan developed with the treating sports dietitian. Particularly when compulsive exercise is a feature of the ED, careful attention should be paid to psychological readiness to return to activity, including any activity that the athlete might have once associated with weight/shape control. Weight targets should be established based on individualized growth history by providers with sport and/or ED/DE expertise. This expertise is particularly important when the athlete may have a growth history that indicates chronic weight suppression (e.g., an athlete who specialized at an early age and might have never reached a BMI percentile that was in the normal range compared to age-matched peers). In these cases, there should be careful consideration and communication with the athlete regarding the benefits of restoration to a weight status that might be higher than they have ever had, in order to promote resumption of menses and muscle and bone integrity, as well as prevent relapse of the ED.

***Evidence-Based Statement***: Current psychotherapy treatments for athletes remain largely untested, but evidence suggests that standard ED care is also appropriate for athletes. ***Grade:*** Level B.

## Diagnostic and Treatment Updates to the Reproductive Function Continuum

### General Comments

The 2014 Female Athlete Triad Coalition Consensus Statement on Treatment and Return to Play included information on screening, risk stratification, and diagnosis of the reproductive components of the Triad [4]. Key advances regarding the reproductive function continuum, addressed in *2025 Update to the Female Athlete Triad Coalition Consensus Statement Part 1: State of the Science and Introduction of a New Adolescent Model* [1] paper, include more specificity as the Triad model now includes a notation on the “pace” of either induction of, or recovery from, menstrual disturbances. Furthermore, new findings have documented the complicating effects of hyperandrogenemia on our understanding of the presentation of FHOA in exercising women [39, 144, 145]. We also address the evidence supporting a reduced risk of reproductive perturbations due to exercise and caloric restriction in women with an older gynecological age [49, 146, 147] and the importance of considering psychosocial stressors in the evaluation of exercising women presenting with Triad conditions [49, 148–155]. Lastly, based on recent RCT data, we now better understand the magnitude of change in dietary energy intake that is causally related to reproductive recovery, and we have characterized the quality of recovery menstrual cycles, i.e., length, presence or absence of ovulation, and luteal phase status [38]. Notably, we now understand that improvements in ovarian steroid exposure can lag well behind clinically recognized improvements in menstrual frequency and regularity [38]. That is, the successful restoration of menses achieved with a yearlong nutritional intervention in exercising women with energy related menstrual disturbances *is not necessarily associated with an overall significant increase in ovarian steroid exposure*, likely due to many recovery cycles being irregular in length and/or anovulatory, and due to a high prevalence of menstrual disturbance relapse [38]. The aforementioned advances in Triad science are summarized herein for clinical application.

### What are the Diagnostic Steps Involved in the Identification of FHOA?

**Fig. 4:**
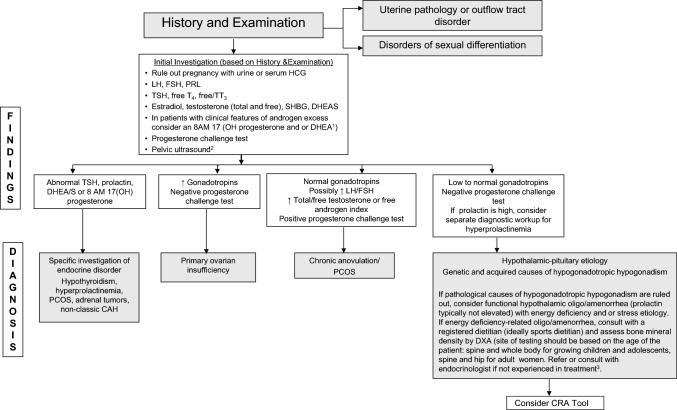
Clinical Evaluation of Primary/Secondary Amenorrhea or Oligomenorrhea. Note: this algorithm is suggested for cis gender adults and adolescents. Recommended clinical evaluation of an athlete with primary or secondary amenorrhea, or prolonged oligomenorrhea, includes a history and physical examination, initial and follow-up laboratory testing and diagnosis by a physician. Referral or consultation with an endocrinologist is recommended if the diagnosing physician is not experienced with treatment of functional hypothalamic oligo/amenorrhea or other etiologies of amenorrhea. *DHEA* dehydroepiandrosterone, *DHEA/S* dehydroepiandrosterone sulfate, *FSH* follicle-stimulating hormone, *hCG* human chorionic gonadotropin, *LH* luteinizing hormone, *PCOS* polycystic ovarian syndrome, *PRL* prolactin, *T4* thyroxine, *TT3* total triiodothyronine, *TSH* thyroid-stimulating hormone, *SHBG* sex hormone binding globulin. Modified from Illingworth with permission [156]. ^1^DHEA may be helpful in diagnosing certain types of adrenal tumors. ^2^To assess intactness of internal genitalia in patients with primary amenorrhea before proceeding with progesterone challenge test, and may be part of a diagnostic work-up for PCOS. ^3^See Table 7

**Fig. 5:**
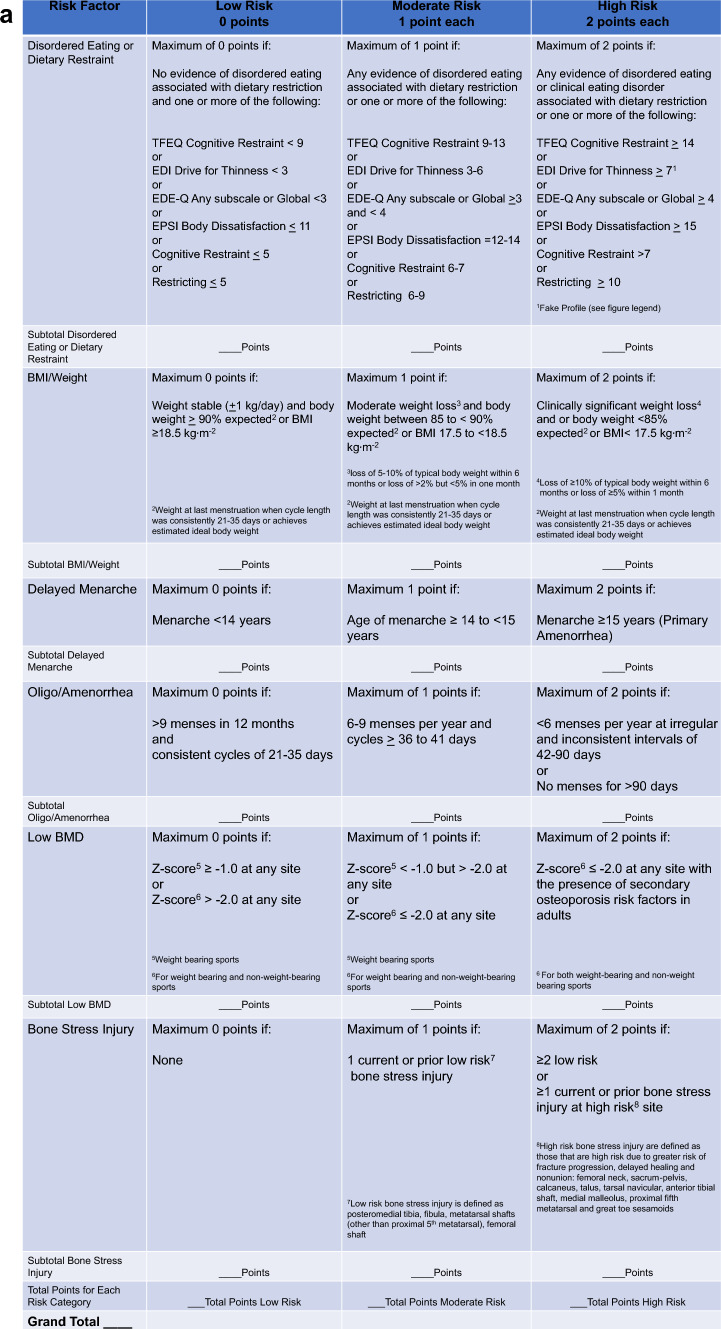

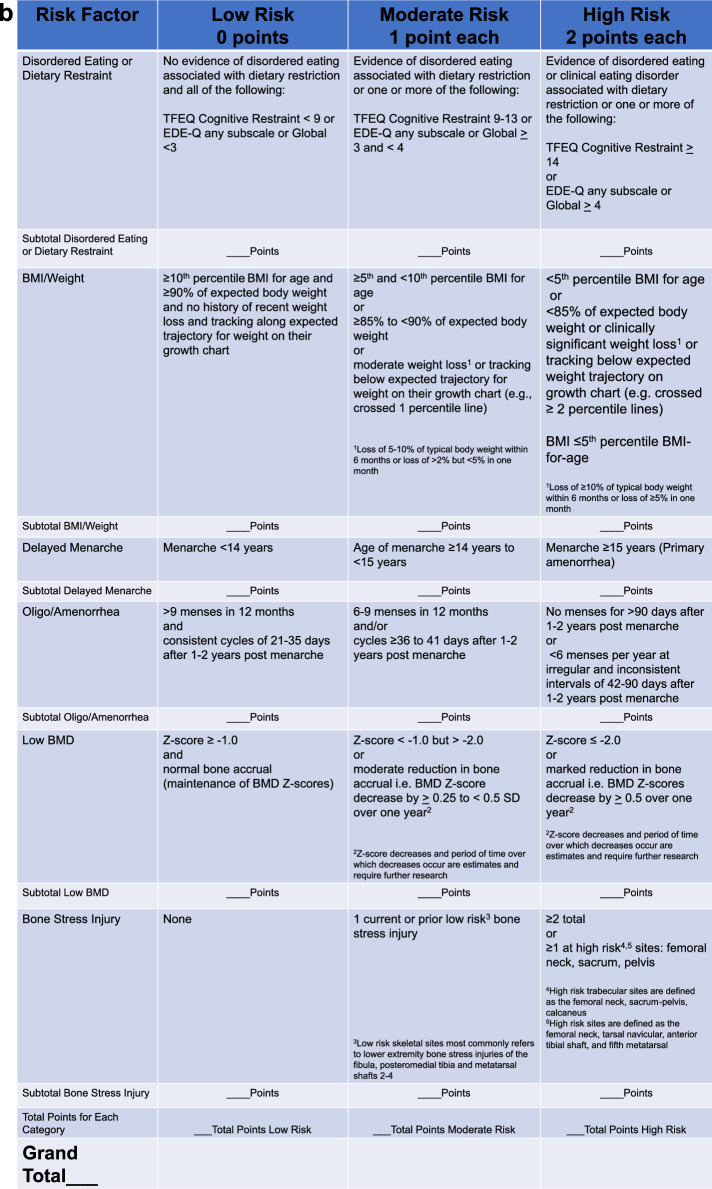
**a** Revised 2025 Adult Female Athlete Triad Cumulative Risk Assessment Tool. The revised cumulative risk assessment tool provides an objective method of determining an athlete’s risk using risk stratification and evidence-based risk factors for the Female Athlete Triad and considers all updates in the 2025 consensus papers. This assessment tool is then used to determine an athlete’s clearance for sport participation (Fig. 6). Points are assigned for each risk factor independently according to the level of risk (low, moderate, high) such that if any of the criteria for a given factor are met, the appropriate points are assigned. A “fake profile” for the EDI drive for thinness score associated with high risk is ≥ 7 or when the scores on the EDI indicate a “fake profile”, a strategy identified by O’Connor et al. 1995 [249] to assign high risk when a drive for thinness score of 0 is in the presence of all other scores < 3 except for a perfectionism score of ≥ 9. This strategy has been used in previous work demonstrating a link between a high drive for thinness, energy deficiency, and menstrual disturbances in exercising women [64]. *BMD* bone mineral density, *BMI* body mass index, *EDI* Eating Disorder Inventory, *EPSI* Eating Pathology Symptoms Inventory, *TFEQ* = Three Factor Eating Questionnaire. **b** New 2025 Adolescent Female Athlete Triad Cumulative Risk Assessment Tool. The cumulative risk assessment tool provides an objective method of determining an athlete’s risk using risk stratification and evidence-based risk factors for the Female Athlete Triad and considers all updates in the 2024 consensus papers. This assessment tool is then used to determine an athlete’s clearance for clearance and return to play (Fig. 6). Points are assigned for each risk factor independently according to the level of risk (low, moderate, high) such that if any of the criteria for a given factor are met, the appropriate points are assigned. *BMD* bone mineral density, *BMI* body mass index, *EDI* Eating Disorder Inventory, *EPSI* Eating Pathology Symptoms Inventory, *TFEQ* Three Factor Eating Questionnaire

**Fig. 6:**
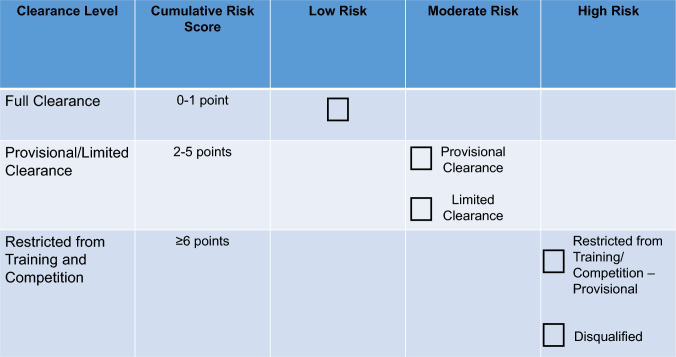
The 2025 Adult and Adolescent Female Athlete Triad: Clearance and Return to Play Guidelines by Medical Risk Stratification. Stratification decisions are based on the cumulative risk score determined by summing the score of each risk factor at the appropriate risk level (low, moderate, high risk) from the Cumulative Risk Assessment Tool (Fig. 5a, b). Clearance and Return to Play status for athletes moderate-to-high risk for the Triad: provisional clearance is at time of evaluation with possibility for status to change over time depending on athlete’s clinical progress; limited clearance is clearance that is granted but with modification in training as specified by a physician with the possibility for status to change depending on clinical progress and new information gathered; restricted from training/competition (provisional) occurs when the athlete is not cleared or able to return to play at the present time with clearance status re-evaluated by a physician and multidisciplinary team with clinical progress; disqualified refers to not safe to participate at the present time with clearance status to be determined at a future date depending on clinical progress, if appropriate. It is the recommendation of the Consensus Panel that athletes diagnosed with anorexia nervosa who have a BMI < 16 kg/m^2^ or with moderate to severe bulimia nervosa (purging > four times per week) should be categorically restricted from training and competition. Future participation is dependent on treatment of their eating disorder, including ascertainment of BMI > 18.5 kg/m^2^, cessation of bingeing and purging and close interval follow-up with the multidisciplinary team

A detailed explanation on how to diagnose FHOA was presented in the 2014 Consensus statement [3, 156]. In brief, since no one test can confirm a diagnosis of FHOA, practitioners must rule out pregnancy and other endocrinopathies to confirm a diagnosis in athletes and exercising women, and as such, FHOA is largely a diagnosis of exclusion. It is also important to rule out the use of medications that might cause oligo/amenorrhea (such as depot medroxyprogesterone acetate, the progestin releasing intrauterine device, high-dose glucocorticoids, gonadotropin releasing hormone analogs, and medications that cause hyperprolactinemia). The specific endocrine conditions that must be evaluated for and ruled out include (1) thyroid dysfunction, (2) hyperprolactinemia, (3) primary ovarian insufficiency, (4) congenital and acquired causes of hypogonadotropic hypogonadism (genetic or non-genetic), (5) hyperandrogenic conditions including polycystic ovary syndrome (PCOS), virilizing ovarian tumors, adrenal tumors, non-classic congenital adrenal hyperplasia and Cushing’s syndrome, and (6) outflow tract obstructions, especially for those women with primary amenorrhea [157, 158]. The 2014 consensus paper [3] presented an algorithm for the diagnosis of primary and secondary amenorrhea. The algorithm has been updated and modified according to previous literature [3, 156] (Fig. 4), illustrating the logical connections among the FHOA diagnostic algorithm and the revised cumulative risk assessment algorithms (Figs. 5a, b, 6).

Primary-care physicians not experienced in diagnosing or treating endocrine conditions should refer to or consult with an endocrinologist to assess reproductive dysfunction. Readers are referred to the Endocrine Society’s Clinical Practice Guideline for the diagnosis of functional hypothalamic amenorrhea [40]. The plan usually includes a thorough medical history, a physical examination, and laboratory and other testing to rule out the endocrinopathies previously mentioned. Laboratory testing should include a pregnancy test (urine or serum) and assessment of levels of thyroid stimulating hormone (TSH), free T4, total and/or free T3, prolactin, follicle stimulating hormone (FSH), luteinizing hormone (LH), total and/or free testosterone, sex hormone binding globulin (SHBG) and dehydroepiandrosterone sulfate (DHEAS). These tests are useful in ruling out pregnancy, primary hypothyroidism, hyperprolactinemia, primary ovarian insufficiency, and PCOS as causes of oligo/amenorrhea. A serum estradiol and/or a progesterone challenge test (medroxyprogesterone acetate 5–10 mg daily for 5–10 days, norethindrone acetate 5 mg daily for 10 days, or micronized progesterone 200–300 mg daily for 10 days) may be useful to assess the severity of hypoestrogenemia, as lack of a withdrawal bleed suggests insufficient estrogenization of the uterine endometrium. In those with primary amenorrhea, the progestin challenge test should be performed after assessing the intactness of the internal genitalia, usually with imaging studies, such as a pelvic ultrasound. If there is physical evidence of significant androgen excess (i.e., hirsutism, acne, androgenic alopecia, clitoromegaly), additional laboratory testing may include an 8:00 a.m. 17-hydroxyprogesterone and dehydroepiandrosterone (DHEA) test. While moderate elevations of DHEA sulfate (DHEAS) may be seen in patients with PCOS, very high concentrations of DHEAS or DHEA are of concern for adrenal tumors. Some tumors are poorly differentiated and lack the enzyme necessary for sulfation of DHEA, and thus both DHEA and DHEAS should be assessed if this is a diagnostic consideration. High concentrations of 8:00 a.m. 17-hydroxyprogesterone are of concern for non-classic 21-hydroxylase deficiency (the most common cause of congenital adrenal hyperplasia). Sometimes, an adrenocorticotropic hormone (ACTH) stimulation test becomes necessary to make this diagnosis. A pelvic ultrasound may be obtained in those with clinical or biochemical hyperandrogenism to confirm polycystic ovaries or to rule out virilizing ovarian tumors [157, 159]. While a pelvic ultrasound may aid in the diagnosis of PCOS in adults, this is not recommended for the diagnosis of PCOS in adolescents because pubertal ovaries in girls even without PCOS may demonstrate a multicystic appearance.

Special attention to clinical factors must be considered when differentiating between oligomenorrhea and amenorrhea secondary to hyperandrogenism rather than energy deficiency and those are highlighted in this update. Factors that discriminate oligomenorrhea and amenorrhea secondary to hyperandrogenism versus energy deficiency include higher BMI, fat mass, upper to lower body fat mass ratio, BMD, LH/FSH ratio, free androgen index (FAI) (FAI = (total testosterone/sex hormone binding globulin) • 100)), and concentrations of insulin, leptin, LH, testosterone, androstenedione, anti-Müllerian hormone, and SHBG [39, 144, 145]. Additionally, oligomenorrhea, in general, has been proposed to be more commonly related to hyperandrogenism than energy deficiency [144]. One study that performed frequent blood sampling for testosterone and pituitary hormones reported that endurance trained women who were oligomenorrheic demonstrated higher testosterone concentrations with no other differences compared to controls, consistent with a hyperandrogenic profile as observed in PCOS, whereas women who were amenorrheic demonstrated reduced LH pulsatility with higher basal growth hormone and cortisol levels, consistent with what is expected in FHOA associated with energy deficiency [160]. Importantly, though, not all women with oligomenorrhea have androgen excess, and in a clinical or research laboratory setting, assessing FAI may be used to screen for hyperandrogenemia. In laboratory research settings, an FAI value of > 4.26 has been used to discriminate between exercising women with hyperandrogenemia and exercising women with menstrual disturbances due to hypothalamic suppression secondary to energy deficiency [39]. The cutoff of > 4.26 represents one standard deviation above the mean FAI value of a reference group (n = 76) of exercising premenopausal women with ovulatory, eumenorrheic menstrual cycles with no luteal phase defects [39]. Menstrual disturbances in exercising women with an FAI ≤ 4.26 are more likely to be caused by energy deficiency than hyperandrogenemia. However, FHOA and hyperandrogenism may co-exist, with the latter becoming more evident with energy repletion.

Proper diagnosis of the etiological basis of menstrual disturbances in exercising women is particularly relevant, as the etiology will determine the recommended course of treatment. In some cases, an increase in energy intake may not be ideal in women with hyperandrogenism, as characteristics associated with hyperandrogenism (i.e., elevated body weight, body fat, and lean mass) may become exacerbated with refeeding. However, data suggest that some women with PCOS and FHOA have lower BMD than controls, so it may still be important to address energy deficiency in these women, as they may still need to gain weight to optimize their BMD [145]. Further, with weight gain, women with hybrid hyperandrogenism and hypothalamic inhibition exhibit *increasing* evidence of hyperandrogenism and irregular menses; some will fail to resume regular menses despite full normalization of weight [145]. For energy-recovered women with persistent menstrual disturbances due to hyperandrogenism or PCOS, the management should focus on preventing endometrial hyperplasia and achieving uterine health through periodic shedding of the uterine endometrium with either a combined estrogen-progesterone preparation or a cyclic progestin, or by inducing atrophy of the uterine lining through the continuous use of such preparations or a progestin-eluting intrauterine device [161–163]. Features of hyperandrogenism benefit from suppression of ovulation and reduction in androgen levels with a combined oral-progestin preparation and androgen receptor blockers, such as spironolactone (a World Anti-Doping Agency (WADA) prohibited substance) or cyproterone acetate [161–163]. A diagnosis of non-classic congenital adrenal hyperplasia may require administration of glucocorticoids to suppress androgen production, and adrenal tumors typically require surgical management. A discussion of how hyperandrogenism complicates the study of menstrual disturbances in exercising women is included in *2025 Update to the Female Athlete Triad Coalition Consensus Statement Part 1: State of the Science and Introduction of a New Adolescent Model* [1].

***Evidence-Based Statement:*** In some exercising women with menstrual disturbances, energy deficiency may co-occur with hyperandrogenemia. ***Grade:*** Level B.

***Evidence-Based Statement:*** Hypothalamic inhibition of the hypothalamic pituitary ovarian axis secondary to energy deficiency may restrain the typical symptoms of hyperandrogenism in exercising women. ***Grade:*** Level B.

### What Roles do Gynecological Age and Psychosocial Stress Play in the Development of FHOA?

As described in *2025 Update to the Female Athlete Triad Coalition Consensus Statement Part 1: State of the Science and Introduction of a New Adolescent Model* [1], a more advanced gynecological age may reduce an individual’s susceptibility to energy deficiency related menstrual disturbances [146, 147]. Resistance to the suppressive effects of energy deficiency in gynecologically mature athletes may represent a factor that modifies a clinician’s assessment of Triad risk. While not well addressed in the Triad literature to date, a greater awareness of the possible protective effect of gynecological maturity, for example, gynecological age ≥ 14 years, against the development and/or severity of Triad conditions is advised.

Another factor that warrants attention on the part of clinicians and sports medicine practitioners is psychosocial stress. Both energy deficiency [26, 27] and psychosocial stress (through its actions on the hypothalamic pituitary adrenal axis and other neuromodulators of gonadotropin releasing hormone activity [164]) are known factors involved in the etiology of FHOA [148, 165–167]. Although a combined effect of metabolic and psychogenic stressors has been documented in the induction of menstrual disturbances in exercising non-human primates [165], little attention has been paid to the clinical relevance of assessing the possible role of psychosocial stressors, and one’s susceptibility to stress, in addition to the role of energy deficiency, in the assessment of Triad risk and/or as a factor in the recovery of an individual with the Triad. While research documenting associations between stress and Triad factors is accumulating [167–169], prospective studies in exercising women have yet to demonstrate an independent effect of stress on menstrual function. That said, an increased awareness on the part of clinicians and sports medicine practitioners of the potential role of psychosocial stressors or one’s susceptibility to stress as a modifier of Triad risk is warranted.

### What is the Definition of Reproductive Recovery?

The concept of “menstrual or reproductive recovery” is not consistently defined in the literature and varied definitions have been utilized [170]. The lack of consistent definitions for menstrual recovery creates a challenge when deciding on endpoints that exercising women presenting with menstrual disturbances should achieve in order to be considered “recovered.” Limiting the definition of recovery to only the occurrence of menses may not align with complete recovery of ovulation and ovarian hormone concentrations. An in-depth discussion on varied definitions of reproductive recovery is presented in *2025 Update to the Female Athlete Triad Coalition Consensus Statement Part 1: State of the Science and Introduction of a New Adolescent Model* [1]. Data from the REFUEL RCT [37, 38, 171, 172] demonstrated the varied reproductive recovery phenotypes in response to a 12-month nutritional intervention of increased caloric intake by 20–40% above baseline energy needs. For summary purposes, Table 4 describes suggested progressive definitions illustrating an increase in the “quality” of reproductive recovery and the degree to which improvements in ovarian steroid exposure can be expected.

**Table 4:** Suggested definitions for quality of reproductive recovery in response to nutritional intervention for FHOA

Definition	Characteristics	Ovarian steroid exposure
Simple recovery	Occurrence of menses	Increase not likely
Definition 2	≥ 1 cycle of < 36 days	Increase not likely
Definition 3	≥ 2 cycles of < 36 days	Increase not likely
Definition 4	≥ 3 cycles of < 36 days	Increase more likely for estrogen and for progesterone if accompanied by ovulation

*FHOA* functional hypothalamic oligo/amenorrhea

***Evidence-Based Statement:*** Clinicians should consider a more comprehensive approach to defining endpoints for reproductive recovery in response to a nutritional intervention including the goal of achieving successive menstrual cycles with lengths of < 36 days and increasing ovarian steroid concentrations. ***Grade:*** Level A.

### What is the Role of Energy Intake in Menstrual Recovery?

In oligo/amenorrheic exercising women participating in a 12-month RCT, REFUEL, a modest increase in daily energy intake (330 ± 65 kcal/day, + 18 ± 4%) for 12 months was sufficient to improve menstrual regularity, which occurred in concert with increased body weight (4.9%), fat mass (18%), and TT_3_ (16%) [37]. Increases in body weight, BMI, fat mass, and percent body fat were positively associated with achieving menstrual recovery. These results support the findings of previous case studies [74, 173, 174], a retrospective study [175], and prospective studies [176–178]. Based on these findings, an increase in energy intake between 300 and 600 kcal/day may be adequate to initiate menstrual recovery [37, 177, 178], which is in agreement with the recommendation in our prior Consensus Statement [2–4]. Notably, the time course to recovery may be longer in women with greater duration of menstrual dysfunction [177].

Although increases in body weight and fat mass have been associated with improvements in menstrual regularity [74, 173, 175, 176], the likelihood of menstrual recovery may also be influenced by baseline body composition prior to the initiation of a dietary intervention. In the REFUEL RCT, the likelihood of experiencing a menses during the intervention of increased energy intake was influenced by body fat at baseline, such that the higher fat mass prior to the intervention, the greater the likelihood that the woman would experience menses during the intervention [37]. An in-depth discussion of the effects of increased energy intake on reproductive recovery is included in *2025 Update to the Female Athlete Triad Coalition Consensus Statement Part 1: State of the Science and Introduction of a New Adolescent Model* [1].

***Evidence-Based Statement:*** A nutritional intervention designed to increase energy intake between 300 and 600 kcal/day will likely *initiate* reproductive recovery. However, factors such as menstrual history, baseline menstrual status, i.e., amenorrhea or oligomenorrhea, baseline body weight, the possibility of relapse, and lifestyle factors such as psychosocial stress are predictors of the timing and success of such interventions. ***Grade:*** Level B.

## Diagnostic and Treatment Updates to Bone Health

### General Comments

Both low energy availability/energy deficiency and suppression of the hypothalamic-pituitary-ovarian axis have been well documented to negatively impact bone health in exercising adolescent girls and young adult women with oligo/amenorrhea, impacting clinically relevant endpoints such as bone turnover [179–182], areal [46, 47, 183] and volumetric bone mineral density (BMD), and bone geometry [36, 44, 47, 184, 185]. This section describes indications for and recommended sites of dual energy X-ray absorptiometry (DXA) assessments, the definition of low BMD and osteoporosis in adolescent and young adult athletes, and non-pharmacological and pharmacological treatment recommendations for impaired bone health.

### Diagnostic Assessment of Bone Health

#### Who Should get a Dual Energy X-Ray (DXA) Scan?

Recommendations for obtaining a DXA scan for BMD testing follow the Triad risk stratification recommendations in exercising adolescent and adult women (Tables 5, 6).

**Table 5:** Guidelines for DXA scanning in adult athletes

**≥ 1 ‘High-risk’ Triad risk factors:** History of DSM-5 [52] diagnosis of a clinical eating disorder associated with dietary restriction Evidence of severe energy deficiency: BMI < 17.5 kg/m^2^ OR < 85% expected body weight^a^ OR clinically significant weight loss of ≥ 10% of typical body weight within 6 months or loss of ≥ 5% in 1 month Menarche ≥ 15 years of age No menses for > 90 days OR history of < 6 menses over the preceding 12 months at irregular and inconsistent intervals of 42–90 days after 1–2 years post menarche Prior BMD *Z* score of ≤ − 2.0 (after at least 1 year from the baseline DXA) OR prior history of osteoporosis Current or prior bone stress injury (1 high risk or ≥ 2 low risk)^b,c^
OR
**≥ 2 “Moderate-risk” Triad risk factors:** Evidence of disordered eating associated with dietary restraint based on self-report or validated surveys Evidence of moderate energy deficiency: BMI ≥ 17.5 kg/m^2^ and < 18.5 OR ≥ 85% to < 90% of expected body weight^a^ OR recent weight loss of 5–10% of typical body weight within 6 months or loss of > 2% but < 5% in 1 month Age of menarche ≥ 14 years and < 15 years Luteal phase defects and/or anovulation AND/OR 6–9 menses per year at intervals of ≥ 36 to < 41 days after 1–2 years post menarche One current or prior bone stress injury (low risk)^c^ Prior *Z* score between < − 1.0 but > − 2.0 (after at least 1 year interval from baseline DXA) for weight-bearing sports OR *Z* score^2^ ≤ − 2.0 for weight-bearing and non-weight-bearing sports
OR
** ≥ 6 months on medications that may impact bone, such as (but not limited to):** Depot medroxyprogesterone acetate Pharmacological doses of glucocorticoids Gonadotropin releasing hormone analogs *Evidence Grade: Level B*

*DXA* dual-energy X-ray absorptiometry, *DSM-5* Diagnostic and Statistical Manual of Mental Disorders, 5th edition [52], *BMI* body mass index

^a^Weight at last menstruation when cycle length was consistently 21–35 days or achieves estimated ideal body weight

^b^High risk bone stress injuries are defined as those that are high risk due to greater risk of fracture progression, delayed healing and nonunion: femoral neck, sacrum-pelvis, calcaneus, talus, tarsal navicular, anterior tibial shaft, medial malleolus, proximal fifth metatarsal and great toe sesamoids

^c^Low-risk bone stress injuries is defined as posteromedial tibia, fibula, metatarsal shafts (other than proximal fifth metatarsal), femoral shaft

**Table 6:** Guidelines for DXA scanning in adolescent^a^ athletes

**≥ 1 ‘High-risk’ Triad risk factors:** History of DSM-5 [52] diagnosis of a clinical eating disorder associated with dietary restriction Evidence of severe energy deficiency: Clinically significant weight loss^b^ or tracking below expected trajectory for weight on their CDC growth chart (e.g., crossed ≥ 2 percentile lines) OR < fifth percentile BMI for age OR < 85% of expected body weight (EBW); EBW = based on 50th percentile BMI for age Menarche ≥ 15 years of age Marked menstrual dysfunction: no menses for > 90 days after 1–2 years post menarche OR < 6 menses per year at irregular and inconsistent intervals of 42–90 days after 1–2 years post menarche Prior *Z* score of ≤ − 2.0 (after at least 6 months from the baseline DXA in adolescents) OR prior history of osteoporosis OR prior history of marked reductions in bone mineral accrual Current or prior bone stress injury (1 high risk or ≥ 2 low risk)^c,d^
OR
**≥ 2 “Moderate-risk” Triad risk factors:** Presence of disordered eating associated with dietary restraint based on self-report or validated surveys OR a history of skipping meals in preadolescents/adolescents Evidence of moderate energy deficiency: Clinically significant weight loss^b^ or tracking below expected trajectory for weight on their growth chart (e.g., crossed 1 percentile line) OR ≥ 5th and < 10th percentile BMI for age OR ≥ 85% to < 90% of expected body weight (EBW); EBW = based on 50th percentile BMI for age Age of menarche ≥ 14 years and < 15 years Current or past history of 6–9 menses over 12 months or cycles ≥ 36–41 days after 1–2 years post menarche 1 current or prior stress reaction or fracture (low risk)^d^ Prior *Z* score between < − 1.0 but > − 2.0 for weight-bearing sports OR * Z* score ≤ − 2 weight-bearing or non-weight-bearing sports OR moderate reduction in bone mineral accrual (after at least 6 months from the baseline DXA in adolescents)
OR
**≥ 1 high- or moderate-risk Triad risk factors AND a significant fracture history (i.e. evidence of osteoporosis), including:** ≥ 2 long bone fractures (non-stress) by the age of 10 years OR ≥ 3 long bone fractures (non-stress) up to 19 years OR a vertebral crush (or compression) fracture Will depend on the likelihood of fracture given the magnitude of trauma (low or high impact) and age at which fracture occurred OR ≥ 6 months on medications that may impact bone, such as (but not limited to): Depot medroxyprogesterone acetate Pharmacological doses of glucocorticoids Gonadotropin releasing hormone analogs *Evidence Grade: Level B*

*DXA* dual-energy x-ray absorptiometry, *DSM-5* Diagnostic and Statistical Manual of Mental Disorders, 5th edition [52], *CDC* Centers for Disease Control, *BMI* body mass index

^a^Interpretation requires the use of adolescent-specific DXA software

^b^Loss of ≥ 10% of typical body weight within 6 months or loss of ≥ 5% within 1 month

^c^High-risk bone stress injury is defined as high risk due to greater risk of fracture progression, delayed healing and nonunion: femoral neck, sacrum-pelvis, calcaneus, talus, tarsal navicular, anterior tibial shaft, medial malleolus, proximal fifth metatarsal and great toe sesamoids

^d^Low-risk bone stress injury is defined as posteromedial tibia, fibula, metatarsal shafts (other than proximal fifth metatarsal), femoral shaft

#### What Sites Should be Screened with a DXA Scan?

The recommendations herein are consistent with those from the International Society of Clinical Densitometry (ISCD) for optimal sites of BMD assessment in adults and adolescents [186], which likely applies reasonably well to athletes. Table 7 depicts the ISCD position statement on the appropriate sites that should be scanned with a DXA. In summary, adult women ≥ 20 years should have weight bearing site(s) scanned, including the lumbar (L1-L4) spine, total hip, and/or femoral neck. If unavailable, the non-weight-bearing radius (33%) can be scanned. For children, adolescents, and adults aged < 20 years, it is recommended to scan the lumbar (L1-L4) spine and the whole body less head. DXA assessments of the hip (in addition to the lumbar spine and whole body less head) may be included in the transition period to adulthood, generally after statural growth is complete [187].

**Table 7:** ISCD position statement recommendations [187] on the appropriate sites that should be scanned with a DXA

**Adult women ≥ 20 years:** Weight-bearing sites (PA lumbar spine (L1–L4), total hip, femoral neck) areal BMD Non-weight-bearing sites, namely radius (33%), if weight-bearing sites cannot be assessed
**Children, adolescents and young women < 20 years:** PA lumbar spine BMC or areal BMD (L1–L4) Whole body less head if possible (otherwise whole body) BMC or areal BMD Adjust for growth delay (with adjustment for height * Z* scores^a^) or maturational delay (with bone age) Use pediatric reference data, and when possible, report height * Z* score adjusted * Z* scores^a^
**Note: BMD** ***Z*** **scores and not T-scores should be reported for all children, adolescents and premenopausal women** ***Evidence Grade: Level B***

*ISCD* International Society of Clinical Densitometry, *DXA* dual-energy X-ray absorptiometry, *PA* posteroanterior, *BMD* bone mineral density, *BMC* bone mineral content

^a^Longitudinal Bone Mineral Density in Childhood study and the Zemel calculator: https://zscore.research.chop.edu/calcpedbonedens.php

Studies have reported low BMD at the spine and the hip in adult athletes with BSI impacting trabecular-rich sites [188]. Among collegiate athletes, those with trabecular-rich BSI had lower spine and total hip BMD than those with cortical-rich BSI [189]. Interestingly, lower hip and radius BMD were reported in those with higher-grade BSI compared with lower-grade BSI. However, in a predictive model for time to full return to sport, whole body BMD was a significant predictor (along with magnetic resonance imaging (MRI) grade of BSI, trabecular-rich vs. cortical-rich site of BSI, and BMI). Among adolescent and young adult athletes, lumbar spine and total body BMD *Z* scores were significantly lower in those with a history of two or more stress fractures than in those with fewer such fractures [44]. Similarly, in adolescent and young adult male athletes, lumbar spine and whole body less head BMD *Z* scores were lower in those with trabecular-rich versus cortical-rich BSI sites [190]. Such data confirm the importance of assessing these sites in DXA evaluations of bone health in youth. However, not all athletes who sustain BSI have low BMD, suggesting that risk factors for BSI are complex and DXA measures might not reliably predict all athletes at risk for BSI.

***Evidence-Based Statement***: We recommend assessing BMD with a DXA scan based on details in Table 7. ***Grade:*** Level B.

#### What is the Definition of Low BMD and Osteoporosis?

The diagnosis of osteoporosis in children, adolescents, and premenopausal women cannot be made on the basis of BMD alone. These recommendations are consistent with those from the ISCD for the definition of low BMD in adults and children [186], as defined in Table 8. In addition, the ACSM recommends characterizing areal BMD for female athletes and exercising women engaged in weight-bearing sports as low if the associated *Z* score is < − 1.0 [191], based on data demonstrating increased BSI risk in those whose BMD *Z* scores are < − 1.0 [32].

**Table 8:** Recommendations for the definition of low bone mineral density in children and adolescents (age 5–19 years) and in premenopausal women

**Recommendations for the definition of low bone mineral density and osteoporosis in children and adolescents (age 5–19 years):** We agree with the ISCD position [187] that the diagnosis of osteoporosis in children and adolescents requires (i) the presence of both a clinically significant long bone fracture history AND low BMC or low BMD OR (ii) ≥ 1 vertebral crush (or compression) fracture A clinically significant long-bone fracture history is one or more of the following: ≥ 2 long-bone fractures by age 10 years ≥ 3 long-bone fractures at any age up to age 19 years We agree with the ISCD position [187] that low bone mass or density for female athletes/exercisers engaged in non-weight-bearing sports is defined as BMC or areal BMD * Z* score ≤ − 2.0, adjusted for age, sex, and body size, as appropriate We suggest that low bone density for female athletes/exercisers engaged in weight-bearing sports be defined as areal BMD * Z* score < − 1.0, adjusted for age and sex (and height as and when appropriate) We agree with the ISCD position [187] that while quantitative CT (QCT), peripheral QCT (pQCT), and high-resolution pQCT (HR-pQCT) provide useful data regarding volumetric BMD ± bone geometry ± microarchitecture, these are considered research tools at this time and should not be used clinically unless appropriate reference data bases exist to facilitate interpretation of the output
**Recommendations for the definition of low bone mineral density in premenopausal women:** We agree with the ISCD position [187] that low bone density for adult female athletes/exercisers engaged in non-weight-bearing sports is defined as areal BMD * Z* score ≤ − 2.0, adjusted for age and sex (and height as and when appropriate) We suggest that low bone density for adult female athletes/exercisers engaged in weight-bearing sports be defined as areal BMD * Z* score < − 1.0, adjusted for age and sex (and height as and when appropriate) We agree with the ISCD position [187] that while quantitative CT (QCT), peripheral QCT (pQCT), and high-resolution pQCT (HR-pQCT) provide useful data regarding volumetric BMD ± bone geometry ± microarchitecture, these are considered research tools at this time and should not be used clinically unless appropriate reference data bases exist to facilitate interpretation of the output ***Evidence Grade: Level B***

*ISCD* International Society of Clinical Densitometry, *BMC* bone mineral content, *BMD* bone mineral density, *CT* computed tomography, *pQCT* peripheral quantitative computed tomography, *HR-pQCT* high-resolution peripheral quantitative computed tomography

Monitoring of DXA measures of BMD is important to assess changes over time, to diagnose evolving low BMD in those with persistent features of the Triad, and resolution of low BMD with non-pharmacological or pharmacological therapy. DXA scans may be repeated every 6–12 months in children and adolescents (given the rapid bone turnover at this age), or every 12–24 months in adults, or when the expected change in BMD *Z* score equals or is greater than the least significant change [186].

### What are the Non-pharmacological Treatment Approaches for Triad-Related Bone Loss?

Successful treatment of athletes and exercising women is contingent on a multidisciplinary approach for recovery from the Triad, including involvement of a treatment team that includes a primary care and/or sports medicine physician, a sports dietitian, and a mental health practitioner. Depending on the individual situation, consultation with an endocrinologist, orthopedic surgeon, psychiatrist, social worker, exercise physiologist, certified athletic trainer, family members, and/or team coach (if applicable) may be helpful. Non-pharmacological measures should constitute initial management in athletes/exercising women with energy deficiency, menstrual dysfunction, and low BMD associated with the Triad. Since the pathway to bone loss involves contributions of both energy deficiency and hypoestrogenism related to FHOA [61], the goal of non-pharmacological treatment is to first reverse energy deficiency and restore normal length, ovulatory menstrual cycles. As stated earlier, it is necessary to consider and rule out other factors contributing to oligo/amenorrhea, such as psychological stress [166], hyperandrogenism [39] or even PCOS [40] prior to considering non-pharmacological approaches.

Appropriate nutrition and weight gain are recommended to address underweight athletes/exercisers and those with menstrual dysfunction of hypothalamic origin [4]. In teenage girls with anorexia nervosa and amenorrhea, it can take 6–12 months of being at a healthy weight before menses resume [192]. Further, in the latter study, the girls weighed on an average about 2 kg more at the time of menses recovery than at the time that they became amenorrheic, and the mean weight at the time of menstrual resumption was over 91% of that expected for age and height.

The duration of time required to observe an improvement in bone density is likely greater than that required for reproductive recovery. However, recent evidence suggests that some recovery of bone may be attainable with appropriate treatment over 12 months [3]. Twelve months of non-pharmacological nutrition therapy appears to impart promising benefits to bone turnover. In exercising women with oligo-amenorrhea who were randomly prescribed an increase in their caloric intake (20–40% above baseline energy expenditure) for 12 months, bone formation index increased by 21% compared to 10% in the control group who maintained their baseline diet and exercise throughout the 12-month investigation (statistically non-significant changes, *p* > 0.05) [193]. Similarly, bone resorption index decreased by 1% with increased caloric intake compared to a 10% increase in the control group, translating to an overall 35% increase in bone balance compared to 9% in the control group (statistically non-significant changes, *p* > 0.05) [193]. Further, it appears that a modest daily increase in energy intake (~ 350 kcal/day) may need to occur for durations exceeding a 12-month time frame to impart improvements to BMD, as neither BMD (whole body, lumbar spine, total hip) nor BMD *Z* scores increased in this trial [41]. In fact, at the hip, BMD actually decreased in spite of weight gain and resumption of menses while BMD in an ovulatory reference group showed no change [41]. Conversely, a greater volume of calorie intake may be associated with more expeditious improvements, though future investigations of this effect are warranted. Of note, other studies have demonstrated positive effects of weight gain and menstrual recovery on bone outcomes over 12 or more months [194, 195]. Miller et al. [194] demonstrated a 3.1% and 1.8% mean annual increase in lumbar spine and total hip BMD, respectively, in women with anorexia nervosa who gained weight and resumed menses, versus a 2.6% and 2.4% decrease in lumbar spine and total hip BMD, respectively, in those who did not gain weight or resume menses. Thus, the time course of non-pharmacological treatment should be at least 6–12 months for menstrual recovery, and 12 months or more to observe skeletal improvement.

Adequate calcium intake is essential to optimize bone mineralization, and adequate 25-hydroxy vitamin D (25(OH)D) concentrations in blood are necessary to optimize calcium absorption from the gut. Athletes should meet at least the recommended dietary allowance of calcium and vitamin D in diet with or without supplements. This amounts to 1000–1300 mg daily of elemental calcium and 600 IUs daily of vitamin D [196]. Further, it is important to assess 25(OH)D and parathyroid hormone (PTH) concentrations in blood and optimize intake depending on these measurements. A high PTH is usually associated with low 25(OH)D concentrations and measurements should be repeated after normalization of 25(OH)D concentrations with vitamin D supplementation. A high PTH level despite normal serum 25(OH)D and calcium concentrations might indicate insufficient calcium intake in the diet. The serum calcium concentration is rarely helpful in assessing vitamin D status as an adaptive increase in PTH will keep this in the normal range in most individuals with mild to moderate vitamin D deficiency. Normal PTH and calcium concentrations may be seen in patients with low 25(OH)D concentration and suggest milder forms of vitamin D deficiency. While the impact of milder forms of vitamin D deficiency on skeletal outcomes is unclear, recommendations are to maintain 25(OH)D levels in the normative range [197].

To date, prospective studies exploring the impact of resistance training and high magnitude loading on bone health of exercising women with energy deficiency and menstrual dysfunction associated with the Triad are lacking. There are concerns that high-impact activity in females with low BMD (± fracture history) may in fact result in new fracture(s) [198] or worsen the state of energy deficiency [199]. Further studies are necessary to determine the impact of weight-bearing programs on BMD and fracture risk in athletes and exercising women with low BMD. Additionally, translational research to identify safe methods for instituting cross-training activities that stimulate bone gain may benefit these athlete populations. However, data to this effect are currently scant. There is also the notion that adequate estrogen may be needed for the osteogenic effects of mechanical loading to be optimal [200], as data have demonstrated poor osteogenic benefits of mechanical loading in chronically amenorrheic athletes or exercising women [46, 47].

***Evidence-Based Statement***: We recommend optimizing weight gain and resumption of menses by reversing energy deficiency (by increasing caloric intake, improving nutrition, and/or decreasing exercise energy expenditure) in exercising adolescents and women with energy deficiency, menstrual dysfunction, and low BMD associated with the Triad. ***Grade:*** Level B

***Evidence-Based Statement***: We recommend a multidisciplinary approach to support the athlete/exerciser and achieve nutritional goals; this should include at least a primary care and/or sports medicine physician, and a registered dietitian, preferably a sports dietitian. Additional consultation may be obtained from a mental health practitioner, an endocrinologist, an orthopedic surgeon, a psychiatrist, an exercise physiologist, a certified athletic trainer, family members and/or the team coach. ***Grade:*** Level A

***Evidence-Based Statement***: We recommend optimizing calcium and vitamin D status. ***Grade:*** Level B

***Evidence-Based Statement:*** We recommend continuing non-pharmacologic therapy for at least 6–12 months before considering other therapies unless bone health deteriorates over this period or new fractures arise. ***Grade:*** Level C

### What are the Recommendations for Hormone Replacement Therapy for Exercising Adolescent and Adult Women with Triad-Related Bone Loss?

#### General Comments

Exogenous hormone replacement therapy has been shown to improve bone outcomes, although route of administration is key to such recovery with estrogen replacement. Although estrogen is primarily considered anti-resorptive, data also suggest bone anabolic effects of estrogen [201, 202]. Despite these effects, multiple RCTs have now demonstrated a lack of efficacy of combined oral contraceptive pills in improving bone outcomes [203–206]. Thus, combined oral contraceptive pills should not be prescribed to improve bone outcomes in FHOA and in fact, bone loss may continue if the energy deficit state persists. Moreover, athletes/exercisers who are using combined oral contraceptive pills for contraceptive purposes should be cautioned that these medications may mask spontaneous menstrual resumption. In contrast to combined oral contraceptive pill usage, physiologic estradiol replacement with the transdermal patch with cyclic progestin has been demonstrated in several studies to have beneficial effects on bone [204, 207–209]. The endogenous gonadal androgens have anabolic and anti-resorptive effects, both direct and via their aromatization to estrogens, but exogenous low-dose testosterone [210] and oral dehydroepiandrosterone (DHEA) [211] do not improve bone health. Recommendations for hormone therapies, other pharmacological treatment strategies, and a formal definition for failed response to treatment are provided in Table 9 and described below.

**Table 9:** Recommendations for hormone therapies, other pharmacological treatment strategies, and a formal definition for failed response to treatment

**1. Recommendations for hormone therapies:** We recommend hormone replacement therapy with estradiol and a progestin if spontaneous menses do not resume after 6–12 months of being at a healthy weight/energy replete status, or after a reasonable trial of nutritional, psychological ± exercise intervention, particularly if bone health deteriorates over this period, or if new fractures occur (consistent with Endocrine Society guidelines [40]) ***Evidence Grade: Level A*** We recommend against using combined oral contraceptive pills to re-establish cyclic menses or improve bone density (consistent with Endocrine Society guidelines [40]) ***Evidence Grade: Level A*** We recommend cautioning athletes/exercisers who are using combined oral contraceptive pills for contraceptive purposes that these medications may mask spontaneous menstrual resumption and are not effective in improving BMD; in fact, bone loss may continue if the energy deficit state persists (consistent with Endocrine Society guidelines [40]). Alternative methods to consider for contraception include progestin containing IUDs or implants although this approach may mask menstrual resumption ***Evidence Grade: Level B*** We recommend using physiologic replacement doses of transdermal 17β-estradiol with a cyclic or continuous progestin (for endometrial protection) as hormone replacement ***Evidence Grade: Level A*** We recommend against the use of androgen replacement therapy or recombinant leptin to reverse hypogonadotropic hypogonadism in exercising women with energy deficiency ***Evidence Grade: Level B***
**2. Recommendations for other pharmacological treatment strategies for the clinical sequelae of the Triad:** We suggest considering pharmacological therapy (other than hormone replacement) in women with energy deficiency and menstrual dysfunction associated with the Triad if they have: BMD * Z* score s ≤ − 2.0 with a clinically significant fracture history AND lack of response^a^ to 6–12 months of non-pharmacological therapy AND hormone replacement therapy (in those with oligo-amenorrhea) OR if estrogen therapy is contraindicated or not indicated (eumenorrheic, normoestrogenic athlete) BMD * Z* scores between − 1.0 and − 2.0 with a clinically significant fracture history and ≥ 1 additional Triad risk factors AND lack of response^a^ to 6–12 months of non-pharmacological therapy AND hormone replacement therapy (in those with oligo-amenorrhea) OR if estrogen therapy is contraindicated or not indicated (eumenorrheic, normoestrogenic athlete) Severe, repeated debilitating fractures and associated morbidity ***Evidence Grade: Level C***

*BMD* bone mineral density

^a^Lack of response to therapy has been defined as: (a) a clinically significant reduction in BMD *Z* scores after 12 months of non-pharmacological therapy or (b) occurrence of new clinically significant fractures during non-pharmacological treatment over the course of 12 months

#### Estrogen Replacement Therapy

Recent evidence has documented the importance of the route of administration of estrogen replacement therapy on bone outcomes. In an RCT conducted in adolescent and young adult normal weight oligo/amenorrhoeic athletes 14–25 years old, improvements in lumbar spine and femoral neck BMD over the course of 12 months were dependent on the route of administration and type of hormone therapy, i.e., the transdermal 17 β-estradiol patch (patch) with estradiol administered in physiologic replacement doses (100 µg daily) with cyclic oral micronized progesterone (200 mg daily for 12 days of each month), or the combined oral ethinyl estradiol contraceptive pill (30 µg ethinyl estradiol with 0.15 mg of desogestrel) (pill) [204]. Specifically, participants treated with the patch demonstrated increases in BMD at the spine (2.75%), femoral neck (5.25%), and total hip (1.85%) compared to those receiving the pill or no treatment [204]. The patch group also demonstrated greater increases in total volumetric BMD (2.4%), cortical area (3.1%) and thickness (0.3%), and trabecular number (5.1%) compared to the pill group [205]. Increases were observed in cortical thickness and cortical volumetric BMD at the distal radius [205].

In another study (not an RCT), improvements in BMD were similarly noted following 12 months of transdermal 17β-estradiol administration in amenorrheic athletes with low body weight [208]. These findings are consistent with RCT data of transdermal estradiol administration in low-weight adolescents with anorexia nervosa, in whom transdermal 17β-estradiol at a 100 µg dose (with a cyclic progestin) improved spine and total hip areal BMD compared to placebo over 18 months [209]. More recently, in older adolescents and young adults with anorexia nervosa, transdermal 17β-estradiol (100 µg daily) with cyclic progestin led to greater increases in areal BMD at the lumbar spine, total hip, and whole body compared to healthy normal-weight controls over 12 months, with greater increases also observed for radial cortical volumetric BMD and failure load [207]. These studies demonstrated that the rate of improvement in bone outcomes was greatest during the first 6 months of therapy and continued at a slower rate over the next 6–12 months [204, 209].

Importantly, in the RCT of transdermal 17β-estradiol versus oral combined ethinyl estradiol and desogestrel versus no treatment in oligo/amenorrheic athletes, the transdermal patch group did not demonstrate the increase in SHBG (binding protein of sex steroids that drives their bioavailability), or suppression of IGF-1 (an important bone-trophic hormone) observed in the oral pill group [205, 212]. Further, the transdermal patch group demonstrated that increased estradiol concentrations noted in this group were associated with increases in BMD at multiple sites. The patch group had a suppression of sclerostin, preadipocyte factor-1, and brain-derived neurotropic factor, all of which otherwise inhibit osteoblastic activity and bone formation [212]. Another 12-month study reported reductions in lumbar spine marrow adipose tissue following transdermal 17β-estradiol administration, associated with improvement in radial cortical volumetric BMD in young women with FHA [213]; in general, higher marrow adipose tissue is associated with lower biomechanical strength of bone. Together, these findings indicate that transdermal estradiol administration improves areal and volumetric BMD, bone geometry, and structure in young oligo/amenorrheic athletes via multiple mechanisms, and that combined oral contraceptive pills should not be used as a method to improve bone health in these athletes given their lack of efficacy. As discussed in the Endocrine Society guidelines for Functional Hypothalamic Amenorrhea [40], combined oral contraceptive pills should also not be used as a strategy to merely resume cyclic menses in this population, as they can mask ongoing energy deficiency-related menstrual disturbances, without demonstrating an improvement in bone outcomes, and they can mask the spontaneous resumption of menses with energy repletion.

In the study examining the impact of estrogen administration on BMD in low-weight amenorrheic athletes [208], greater increases in areal BMD were observed in athletes who spontaneously resumed menses compared to those receiving transdermal estradiol therapy or no therapy [208]. This is likely because spontaneous resumption of menses typically requires an improvement in body weight and energy status, which in turn should result in an improvement in body composition, metabolic hormones, and gonadal steroid hormone concentrations induced by energy deficiency. For instance, improvements in energy status may result in increased lean mass, reduced marrow adipose tissue, increased IGF-1 and leptin, reduced cortisol and peptide YY, and increased estrogen and other gonadal steroid concentrations, all of which should favorably impact bone [214].

It is important to note that a progestin should be administered either cyclically or continuously in athletes receiving continuous transdermal 17β-estradiol to prevent endometrial hyperplasia, which can increase the risk of endometrial cancer in the long-term. Options include micronized progesterone (200 mg daily for at least 12 days of every month), medroxyprogesterone acetate (5–10 mg daily for 10 days every month), norethindrone acetate (5 mg daily) or a progestin releasing intrauterine device (IUD) [40]. It is important to caution athletes that the combination of transdermal estradiol and a cyclic progestin has no contraceptive efficacy, and contraceptive therapy should be employed by athletes who are sexually active if pregnancy prevention is desired. A progestin releasing IUD has the advantage of providing protection against endometrial hyperplasia in those using physiologic transdermal estradiol, while also having contraceptive efficacy and no negative impact on bone health [215, 216].

The duration of hormone replacement therapy needs to be determined based on evolution of energy status over time. A trial of hormone replacement may be considered after 6–12 months of non-pharmacological therapy.

#### Gonadal Androgen Replacement Therapy

FHOA is also associated with deficits in gonadal androgen levels, which also have an impact on bone. While data are lacking for the use of androgen replacement in amenorrheic female athletes, one RCT in adult women with anorexia nervosa of transdermal testosterone replacement (versus placebo), to maintain testosterone levels in the upper half of the normal range for women, did not demonstrate a significant effect of testosterone on areal BMD over 1 year [210]. In contrast, in another RCT, the combination of 50 mg of oral micronized dehydroepiandrosterone (a gonadal and adrenal steroid) with a combined oral contraceptive pill containing 20 µg of ethinyl estradiol and 0.1 mg levonorgestrel, resulted in maintenance of areal BMD *Z* scores in young women with anorexia nervosa aged 13–27 years [217]. However, a subsequent study in adolescents with anorexia nervosa 12–18 years old using this combination reported no difference from placebo in girls who had closed epiphyses and had completed growth, and a deleterious impact on areal BMD measures in those with open epiphyses who were still growing [218].

#### Leptin Replacement Therapy

Leptin stimulates kisspeptin secretion, and therefore gonadotropin releasing hormone pulsatile secretion, and is also bone anabolic. In a small 3-month trial of metreleptin versus placebo in adult women with FHOA, women who received metreleptin demonstrated improved menstrual function and an increase in surrogate markers of bone formation, IGF-1, IGFBP-3, and the thyroid hormones [219]. However, consistent with the anorexigenic effects of leptin, the metreleptin group reported a subjective reduction in appetite, and sustained a significant reduction in body weight—not an optimal outcome in FHOA associated with energy deficiency. In a subsequent small 9-month RCT of metreleptin versus placebo in which metreleptin levels were adjusted to prevent weight loss, women who received metreleptin had a significant improvement in lumbar spine bone mineral content but sustained a reduction in fat mass (a marker of energy stores) despite careful dose adjustments [220]. As such, leptin therapy does not appear to be a viable strategy to address bone health in women with energy deficiency and menstrual dysfunction associated with the Triad.

#### Osteoanabolic Therapies

Limited data are available regarding the use of osteoanabolic drugs such as recombinant PTH, abaloparatide (PTHrP analog), and romosozumab (anti-sclerostin antibody) to improve bone health in women with energy deficiency and/or FHOA. Romosozumab also has antiresorptive effects on bone. Most of these studies have been reported in women with anorexia nervosa with some overlap with the Triad. However, studies in women with the Triad are currently lacking.

A small 6-month RCT of teriparatide versus placebo in older pre-menopausal women with anorexia nervosa and FHOA reported significant increases in spine (but not hip) BMD with teriparatide [221]; longer-term studies and studies in athletes assessing the impact on BMD are currently lacking. There are some data (mostly case reports and case series) suggesting that teriparatide may promote fracture healing. However, these data are not definitive. Further, no studies have reported on the impact of abaloparatide or romosozumab on bone outcomes in energy deficiency and/or FHOA. One retrospective case series has recently demonstrated significant improvements in bone density associated with reductions in bone turnover 6 and 12 months following administration of romosozumab in women with anorexia nervosa and severe osteoporosis [222]. An RCT of romosozumab versus placebo in women with anorexia nervosa is currently ongoing and results from this study should inform management of FHOA with this osteoanabolic and anti-resorptive agent [223].

IGF-1 is a bone anabolic hormone that is typically low in conditions of energy deficiency, and some studies have examined the impact of IGF-1 replacement in women with anorexia nervosa. One 9-month RCT reported an increase in spine and hip BMD in women with anorexia nervosa who received a combination of recombinant human IGF-1 (rhIGF-1) in replacement doses and an estrogen-progestin combination pill versus those who received neither [224]. However, another RCT in which adolescents and young adult women with anorexia nervosa receiving the transdermal estradiol patch with cyclic oral progesterone were randomized to rhIGF-1 in replacement doses or placebo for 12 months reported no additional benefits of rhIGF-1 beyond those observed with transdermal estradiol and cyclic progesterone alone [225].

Based on the available literature, the evidence to support osteoanabolic therapies for bone health in exercising women with energy deficiency and menstrual dysfunction associated with the Triad is insufficient to support definitive recommendations.

#### Bone Anti-Resorptive Therapies

Data are also limited for the role of antiresorptive drugs in improving bone health in women with energy deficiency and FHOA associated with the Triad. In adult women with anorexia nervosa, one 12-month RCT reported a significant increase in spine and hip BMD following administration of risedronate (vs. placebo) [210]. Further, sequential therapy with rhIGF-1 for 6 months followed by risedronate for 6 months (vs. 12 months of risedronate or double placebo) led to greater increases in spine areal and volumetric BMD than in the double placebo group, and greater increases in lateral spine areal BMD than both other groups [226]. However, a 12-month RCT of alendronate versus placebo reported no increase in spine BMD in adolescents with anorexia nervosa, although small increases were noted at the femoral neck [227]. Definitive data for denosumab are currently lacking in this population. However, a small proof of concept study in adult women with anorexia nervosa reported a decrease in bone turnover and an improvement in lumbar spine BMD over 12 months with denosumab given at a dose of 60 mg subcutaneously every 6 months [228].

As such, based on the available literature, there is insufficient evidence to routinely support the use of anti-resorptive therapies for bone health in exercising women with energy deficiency and menstrual dysfunction associated with the Triad. However, bone anabolic or anti-resorptive therapies may be considered as a last resort either alone or in combination in exercising women with energy deficiency who fail therapy with non-pharmacological therapy as well as hormone replacement therapy, or if they have contraindications to estrogen, or are eumenorrheic and normoestrogenic, meaning that estrogen replacement is not indicated. These therapies are also a consideration in those with frequent debilitating fractures and associated morbidity. For the last two groups, it is important to refer the women to a specialist for metabolic work-up to determine the cause of bone compromise.

## Cumulative Risk Assessment for Clearance and Return to Play: Assessment Tool and Guidelines

### General Comments

In 2014, the Female Athlete Triad Coalition Consensus Statement on Treatment and Return to Play of the Female Athlete Triad introduced a standardized risk stratification protocol and guidelines for clearance and return to play [4]. The purpose of the Coalition’s Cumulative Risk Assessment (CRA) tool is to adequately assess, manage or treat female adult and adolescent athletes with the Triad, and it is described in Figs. 5a, b and 6. The CRA should ideally be used at the time of the PPE and administered by a healthcare practitioner (physician, physician assistant, or nurse practitioner). It was developed based on evidence that female athletes with greater cumulative risk factors for the Triad were at increased risk for unfavorable outcomes of low BMD and/or BSI [32, 229] and it incorporates evidence-based risk factors for the Triad and the magnitude (or severity) of risk. The corresponding point scheme is then translated into clearance and return to play guidelines. The evidence-based CRA tool and associated guidelines have since been validated in prospective studies and are demonstrated to be predictive of BSI in female athletes [56, 230–232]. The CRA tool has also been validated in male athletes based on an adapted CRA tool, which removed the menstrual-related categories of delayed menarche and oligo/amenorrhea for application in men [233]. Updated details of the Triad CRA studies and advancement in the recommendations for the use of the CRA tool for appropriate screening at the time of the pre-participation physical and other assessments involved in returning athletes to play are outlined below.

Additional areas of advancement since the previous Female Athlete Triad Consensus Statement was published include: (1) an updated Triad CRA tool including one for adolescents with added flexibility to facilitate its broader implementation during the risk stratification process, (2) inclusion of prognostic factors for BSI, such as type of BSI (cortical-rich or trabecular-rich bone), anatomic site (low- or high-risk location), and MRI grading that correlate with return to play, and (3) special attention focused on the adolescent athlete and unique considerations that relate to injury risk. These advancements are detailed below.

### How Can Risk Stratification be Used to Evaluate Health and Participation Risk?

Risk stratification for female athletes can occur during the PPE or any point when concerns about Triad conditions are noted and decisions about competition and/or return to play are necessary [2, 4]. The Triad CRA includes the following six items with specific definitions scored on a scale from 0 to 2: disordered eating/dietary restraint, BMI/weight, delayed menarche, oligo/amenorrhea, low BMD, and bone stress injury. The resulting CRA score could then be used to assign an athlete to low-, moderate-, and high-risk categories, which would then correspond to a clearance or return to play decision.

Since its introduction, the CRA tool has been tested for its association with impaired bone health. Specifically, a study of 239 collegiate athletes across various sports identified that 29% were classified as having moderate or high Triad CRA scores and had a 2.6 and 3.8 greater likelihood of sustaining a BSI, respectively, compared with women in the low-risk category [230]. Furthermore, the oligomenorrhea/amenorrhea and prior stress fracture/reaction scores were identified as independent predictors for subsequent BSI [230]. In national/world class female distance athletes, women classified in the high-risk category for Triad risk factors had a greater number of all-time fractures (not just BSIs) than the low-risk group [56]. In a 4-year prospective study, approximately 75% of female runners were classified as moderate or high-risk, with each 1-point increase in the Triad CRA score associated with a 13% increased risk for BSI (95% CI: 1–26% increase in risk) [232]. Those athletes belonging to the higher-risk categories also had a significantly increased risk of sustaining a subsequent BSI. Results from these investigations are consistent with other studies that demonstrate that an increase in cumulative risk factors for the Triad is associated with a dose–response relationship with bone health, leading to low BMD and BSI [32, 229, 234, 235] and suggesting that Triad CRA scores are predictive of fracture in female athletes and can help identify athletes at increased risk for BSI.

### Updates to the Triad Cumulative Risk Assessment (CRA) Tool

Despite evidence supporting that the Triad CRA effectively indicates risk for BSI among exercising women [230, 231], there remain barriers to its implementation under certain circumstances that can result in the exclusion of individuals from assessment and/or require alternative methods of implementation [18, 230, 236]. The incorporation of well-defined scoring criteria and flexible methods for implementing the Triad CRA tool may improve the risk stratification process among exercising and physically active women. Specific updates and areas of future investigation are described below.

#### Identifying Dietary Restraint

The original Triad CRA tool included the risk factor of “Low energy availability with or without disordered eating/eating disorder,” which was scored based on the diagnosis of an ED, the presence of DE, and dietary restriction. Although the DSM-5 criteria for ED diagnoses are well-defined, it remains unclear what would constitute DE and/or dietary restriction, particularly for athletes who may have specific nutritional needs. Previously, dietary restraint has been operationally defined for application with the Triad CRA tool [18] based on DE questionnaires that have been related to Triad sequelae [32, 63–65, 229], including the Three Factor Eating Questionnaire (TFEQ) [237] and Eating Disorder Inventory (EDI) [238]. For the TFEQ, a cognitive dietary restraint subscale of < 9 was implemented as indicative of low risk, 9–13 as moderate risk, and > 13 as high-risk. For the EDI, a drive for thinness subscale < 3 was identified as low risk, 3–6 as moderate risk, and > 6 as high risk. Additionally, a “fake” profile, defined as a drive for thinness score of 0 in the presence of all other scores < 3 except for perfectionism of ≥ 9, would also indicate high risk. Notably, other DE questionnaires relating to energy deficiency and risk for the Triad have been implemented and may be applicable for use with the Triad CRA, although specific scoring criteria need to be proposed and tested. Additional questionnaires that may have utility in populations of physically active women to identify dietary restriction include the Eating Disorder Examination Questionnaire (EDE-Q) and Eating Pathology Symptoms Inventory (dietary restraint subscale) (EPSI). Specific scoring criteria that can be incorporated into the CRA for adults are outlined in Table 10 and described here. Furthermore, this risk factor has been renamed to “Dietary Restraint” to better reflect the underlying assessment criteria.

**Table 10:** Female athlete Triad cumulative risk assessment tool scoring criteria for TFEQ, EDI, EDE-Q, and EPSI

	TFEQ	EDI	EDE-Q	EPSI
**Low risk** **0 points**	Cognitive restraint ** < 9**	Drive for thinness ** < 3**	Any subscale or global ** < 3**	Body dissatisfaction ** < 11** Cognitive restraint ** < 5** Restricting ** < 5**
**Moderate risk** **1 point**	Cognitive restraint **9–13**	Drive for thinness **3–6**	Any subscale or global ** > 3 to < 4**	Body dissatisfaction **12–14** Cognitive restraint **6–7** Restricting **6–9**
**High risk** **2 points**	Cognitive restraint ** ≥ 14**	Drive for thinness ** ≥ 7** Fake profile^a^	Any subscale or global ** ≥ 4**	Body dissatisfaction ** ≥ 15** Cognitive restraint ** ≥ 7** Restricting ** > 10**

*EDI* eating disorder inventory, *EDE-Q* eating disorder examination questionnaire, *EPSI* eating pathology symptom inventory, *TFEQ* three-factor eating questionnaire

^a^Fake Profile, to assign high risk when a drive for thinness score of 0 is in the presence of all other scores < 3 except for a perfectionism score of ≥ 9 [249]

The EDE-Q is a validated 28-item questionnaire for use when screening for EDs [239] and has been implemented in Triad research to identify dietary restraint as a risk factor for impaired bone health [32, 229]. The EDE-Q assesses the four subscales of weight concern, shape concern, eating concern, and dietary restraint, as well as a global score (sum of all subscales divided by the number of subscales). Responses are scored on a range from 0 to 6 to reflect the number of days in the previous 4 weeks (28 days) that a respondent experienced each attitude, feeling, or behavior. An EDE-Q dietary restraint score ≥ 3 was chosen to identify an elevated value [32, 229]; however, to also incorporate other eating attitudes and behaviors that may contribute to energy deficiency, the current CRA tool incorporates scoring for all subscales including the global score. An EDE-Q global score (average of the subscales) ≥ 4.0 score indicates high risk and such scores fall within the 80–95th percentiles compared to reference norms [239–244]. While the referenced dietary restraint values in the CRA (i.e. EDE-Q dietary restraint ≥ 3.0) have been associated with low BMD and low EA in athletic populations, individuals with lower EDE-Q scores (< 3.0) may also be at risk for energy deficiency, as suggested from normative data from a community sample of young women and ED populations [239, 245, 246].

The EPSI is a 45-item self-report measure of eating pathology designed for use in diverse populations, to include females and males across a range of body weight categories. Although its use as a proxy indicator of energy deficiency is limited to date, it has been used to assess eating attitudes in physically active populations [247] and discussed as part of the Male Athlete Triad Consensus Statement [248]. The EPSI includes subscales similar to what has been previously applied in Triad research and practice, including body dissatisfaction (dissatisfaction with one’s weight/shape), cognitive restraint (efforts to limit or avoid eating), and restricting (concrete efforts to avoid or reduce food consumption), as well as additional subscales that are not typically included in traditional inventories, such as excessive exercise (exercise that is intense or compulsive) and muscle building (desire for increased muscularity or supplement use) that may be applicable to understanding energy status in exercising and physically active populations. Consistent with the rationale for determining values for the TFEQ and EDI (including the use of the “fake profile” for the drive for thinness score [63, 249]), the scoring criteria suggested for the EPSI are noted in Table 10. For example, moderate risk is above the mean for a healthy population; high risk is similar to the average for an ED population [250].

As the CRA tool is only useful when implemented completely and correctly, the time and resources required for each questionnaire must also be considered when assessing the presence of dietary restraint, DE, and/or an ED. For example, clinical interviews require trained personnel and are time consuming, which may limit their implementation as part of the risk stratification process. Additionally, the EDI-3 requires purchasing of questionnaire booklets and a professional manual that provides diagnostic criteria and clinical norms, which may be cost prohibitive in many settings. Alternatively, the EDE-Q and EPSI questionnaires and scoring systems are freely available. Improving the accessibility of the CRA tool as a whole, and each of the associated individual risk factors, by incorporating free or cost-effective tools during the risk stratification process is an important consideration for improving health equity among exercising women and athletes.

#### Assessment of Energy Deficiency in the Absence of Voluntary Dietary Restriction

Notably, energy deficiency can develop without the presence of DE or an ED. For example, inadvertent undereating occurs when caloric intake does not meet energy expenditure needs in the absence of conscious restriction of food intake, such as due to food insecurity and or logistical reasons [49], and would therefore not be evident based on the aforementioned eating behavior questionnaires. As such, methods of identifying energy deficiency without the presence of DE behaviors are also warranted when identifying women with, or at risk for, the Triad. If resources allow, objective physiological indicators of energy deficiency, such as low mRMR/pRMR ratio or concentrations of TT_3_ [73], may be beneficial for such situations to identify the presence of energy deficiency and/or identify those at risk for impaired bone health. For example, in a sample of exercising women, those who were categorized as “Provisionally Cleared” (i.e., 2–5 CRA points) had significantly lower TT_3_ concentrations than the “Full Clearance” group and ratios of mRMR/pRMR that were indicative of current energy deficiency [236]. Interestingly, the “Restricted Clearance” group did not differ on average, for TT_3_ or RMR ratios compared to the “Full Clearance” group, which may reflect the contribution of historical or non-modifiable factors, such as history of clinical ED, delayed menarche, oligo/amenorrhea, or previous BSI, that may not reflect an individual’s current health status. It is proposed that women who present with objective physiological criteria of metabolic compensation consistent with prolonged energy deficiency should, at minimum, be categorized as “Provisionally Cleared” and subsequently undergo a full clinical workup to include evaluation for other Triad components.

The LEAF-Q was also developed as a screening tool for the identification of female athletes at risk for the Triad [50] and has been interpreted incorrectly and implemented as a surrogate measure of low EA in female athletes [251]. Despite being developed to classify current EA and/or reproductive function and/or bone health, it may not actually be appropriately assessing current energetic status. Validation of the LEAF-Q was dependent on a median split of calculated EA, which is prone to errors in free-living individuals [252], rather than physiological indicators of metabolic compensation (e.g., TT3, RMR). Additionally, despite having utilized EA as a discriminator of positive and negative Triad risk, groups considered at risk and not at risk based on LEAF-Q scores were reported to have a similar EA [50]. There were also no significant differences between positive and negative Triad risk groups in the number of subjects diagnosed with low BMD, DE and ED, and low BMI, which are well-established, evidence-based conditions associated with the Triad. Additionally, menstrual dysfunction and/or impaired bone health may not align with current energetic status as systems may adapt in varying timeframes. It is therefore our suggestion that the LEAF-Q *not* be used, on its own, to identify female athletes at risk for the Triad. Alternatively, a more recent questionnaire to identify exercising women at risk for energy deficiency, the FED-Q, has been developed and has been validated against TT3, a circulating biomarker of energy deficiency [51].

#### Introduction of the CRA for Adolescent Athletes

The CRA for adolescents includes the same risk factors as the adult CRA tool with criteria specific to characterizing adolescent development, changes in weight, height, reproductive function and bone health. The tool includes considerations for bone mineral accumulation, growth trajectory, and time needed for adolescents to establish menstrual regularity after the onset of menses. The definition of osteoporosis is specific to adolescent athletes. For the “Disordered eating associated with energy deficiency” risk factor, surveys with adolescent-specific data are listed, including the EDE-Q, a validated DE/ED assessment, which has exhibited associations with Triad outcomes in cross-sectional and prospective studies of adolescents [240, 243, 253–255]. The TFE-Q has also been used to classify adolescent athletes with elevated dietary restraint and associated Triad outcomes [253].

#### Substitutions for Missing Risk Stratification Categories

**Fig. 7:**
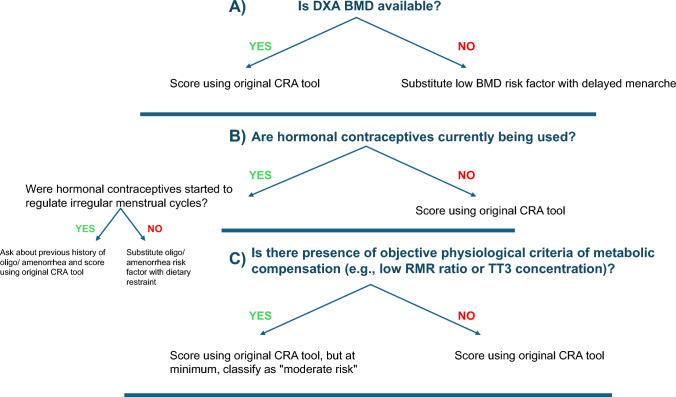
The 2025 Substitutions for the Adult Female Athlete Triad Cumulative Risk Assessment Tool. Evidence-based substitutions are recommended if DXA derived BMD is not available, if hormonal contraceptives are being used, or if there is objective evidence of metabolic compensation. *BMD* bone mineral density, *CRA* cumulative risk assessment tool, *DXA* dual X-ray absorptiometry, *RMR* resting metabolic rate, *TT3* total triiodothyronine

While the Triad CRA tool incorporates evidence-based risk factors for impaired bone health, there are circumstances in which accurate assessments of individual risk factors, such as current menstrual status and BMD, may not be available. For situations when data on individual risk factors may be missing, an alternative scoring system has been proposed [236] and is described below, and a decision tree is presented for ease of use in Fig. 7.

Hormonal contraceptive use is common among women of childbearing age and prevents an accurate assessment of current menstrual status. As frequency of menses is the basis for assessing the “Oligomenorrhea and/or Amenorrhea” risk factor of the Triad CRA tool, additional guidance is necessary for how the risk stratification tool can be implemented for those in whom menstrual frequency cannot be ascertained. First, past history of menstrual frequency (before starting hormonal contraceptive therapy) should be assessed in the context of training and energy status at that time and can be used to score the “Oligomenorrhea and/or Amenorrhea” risk factor as originally described. Additional information that can be helpful is to understand why an individual may be using hormonal contraceptives, i.e., was it for the purpose of restoring absent menstrual cycles? If there is no past history of oligo/amenorrhea, an alternative approach can be to substitute other risk factors that are able to be scored for the missing risk factor, e.g., menstrual history. For example, Koltun et al. [236] demonstrated that in a large sample of exercising women, substituting the “Low Energy Availability” risk factor score, now referred to as “weight/energy status,” in place of the “Oligo/Amenorrhea” risk factor did not significantly change the proportion of women who were categorized in each clearance decision (i.e., low, moderate, high risk).

Similar to “oligo/amenorrhea,” the “low BMD” risk factor can be difficult to assess or may be missing in situations where risk stratification is necessary. Access to DXA is unavailable in many circumstances because of its cost, radiation exposure, and expertise required for its use. When BMD data are not available, similar to what was proposed in cases of hormonal contraception use, a scoring substitution is recommended. In the same large database of exercising women, when the “Delayed Menarche” risk factor score was substituted for the unavailable “Low BMD” score, the proportion of women who were categorized in each clearance decision remained similar [236]. While these strategies may improve the accessibility of the Triad CRA tool in a broader range of women, additional work is required to validate these recommendations and further refine the Triad screening process.

#### Additional Considerations

Although the CRA has been tested for its association with impaired bone health, there have been variations in its exact implementation, and updates to the tool have been made based on more recent research. Additional work must be undertaken to validate the proposed updates and continue to assess the utility of novel and established screening methods.

While not currently part of the Triad CRA tool, there are additional factors that should also be considered when screening for the Triad. For example, energy deficiency exists more commonly in athletes in endurance sports and sports emphasizing leanness (i.e., endurance, aesthetic, weight class) and therefore, these athletes should be regularly screened for Triad risk. One study in female collegiate athletes found that cross-country, gymnastics, and lacrosse teams had the highest proportion of athletes in the moderate- and high-risk categories [230]. Such findings are consistent with evidence that female athletes and active women in lean sports have a higher prevalence of individual and combined components of the Triad compared with non-lean sports [256]. Athletes that participated in lean sports were three times more likely to demonstrate low BMD, defined as *Z* score < − 1.0, compared to non-lean sport participants [229] and while participation in a leanness sport alone was not a risk factor for BSI, it was significant when combined with other risk factors such as dietary restraint and exercising more than 12 h/week [32]. Alternatively, high-volume exercise (defined as ≥ 12 h/week to reflect the minimum training requirements for competitive athletes) was independently associated with increased risk for BSI [32]. However, the original investigation could not identify whether the association between training volume and BSI risk was due to effects on energetic status or biomechanical factors, such as increased bone strain. Also absent from the CRA is a family history of osteoporosis. There is a significantly increased risk of developing osteoporosis if either biological parent has a history of osteoporosis [257].

***Evidence-Based Statement:*** There is a dose–response relationship with Female Athlete Triad risk factors and bone in that the higher the number of cumulative risk factors, the greater risk for low BMD and/or BSI risk. **Grade:** Level B.

***Evidence-Based Statement:*** The Triad Coalition’s CRA score is predictive for BSI in female athletes. **Grade:** Level B.

***Evidence-Based Statement:*** It is proposed that women who present with objective physiological criteria of metabolic compensation consistent with prolonged energy deficiency be categorized, at minimum, as “Provisionally Cleared”. **Grade:** Level C.

***Evidence-Based Statement:*** Female athletes in lean sports have a higher prevalence of Triad risk factors than those in non-lean sports. **Grade:** Level B.

### What other Prognostic Factors can be used for Return to Play Decisions?

Prognostic factors for BSI that correlate with return to play and are involved in healing and recovery should be included in addition to evidence-based screening tools to develop a comprehensive strategy for clearance and return to play recommendations for the female athlete. These prognostic factors include MRI grading of BSI [189, 258–261], type of BSI (trabecular-rich or cortical-rich) [189, 231, 262] as well as anatomic site [263–265].

#### MRI Grading

Fredericson et al. [258] demonstrated that an MRI grading system for BSIs was correlated with the severity of injury [258]. This grading system is still widely used today and is shown in Table 11. A recent systematic review and meta-analysis of MRI grading of BSI and return to play demonstrated that higher grade injuries by MRI (grades 3 and 4) were indeed associated with a longer to return to play than lower grade BSI (grades 1 and 2) [260], consistent with prior prospective studies in collegiate runners [189] and retrospective studies assessing return to play from BSI in a variety of collegiate sports [259].

**Table 11:** MRI grading scale for bone stress injuries (Fredericson et al. [258])

MRI grade	
1	Mild to moderate periosteal edema on T2^a^ Normal marrow edema on T1 and T2
2	Moderate to severe periosteal edema on T2^a^ Marrow edema on T2 but not T1
3	Moderate to severe periosteal edema on T2^a^ Marrow edema on T1 and T2
4	Moderate to severe periosteal edema on T2^a^ Marrow edema on T1 and T2 Fracture line present

*MRI* magnetic resonance imaging

^a^Periosteal edema may not be a necessary criterion in all stages with other lower extremity skeletal sites [258]

***Evidence-Based Statemen****t:* BSIs of higher MRI grades are associated with a delay in return to play compared to BSIs of lower MRI grades. ***Grade:*** Level B.

#### Bone Type

The Triad CRA score is significantly related to trabecular and cortical-rich BSI [231]. In a recent prospective study, each 1-point increase in Triad CRA score was associated with a significant 26% increase in risk of trabecular-rich BSI and a statistically non-significant (*p* = 0.054) 14% increased risk of cortical-rich BSI. Of the CRA scores, “low BMD” and “oligomenorrhea/amenorrhea” risk scores were most predictive of trabecular-rich BSI, and of the DXA measurements, low femoral neck *Z* scores were most predictive of trabecular-rich BSI [266]. Prior studies have also demonstrated that low BMD, BMI, and weight are significantly related to trabecular-rich BSI [188, 189, 267]. Studies have found that the distribution of BSIs in the Triad low-risk category group were primarily in cortical bone compared with the Triad moderate- and high-risk category groups that had a large number of BSIs in bones with greater trabecular composition, including the pelvis and femoral neck [189, 262, 268]. Given the strong association between Triad-related risk factors and BSIs, particularly in trabecular-rich bone, clinicians should ensure adequate screening and management of these health issues to prevent BSIs. Additionally, given that trabecular-rich bone is more metabolically active and sensitive to nutrition and hormone aberrations contributing to lower bone mass, a recent 7-year prospective study suggests that a team-based nutrition intervention program focused on optimizing EA and including individualized nutrition sessions may effectively reduce BSIs, particularly in trabecular-rich locations [268].

***Evidence-Based Statement:*** The Triad CRA tool score is positively associated with the risk of BSI of trabecular-rich skeletal sites. ***Grade:*** Level B.

***Evidence-Based Statement:*** Nutrition intervention programs may reduce BSI, especially in trabecular-rich skeletal sites. ***Grade:*** Level B.

#### Skeletal Site

In addition to risk stratification of BSI by bone type (trabecular-rich and cortical-rich bone), several studies have demonstrated a correlation in bony healing and return to play with skeletal site of BSI and whether it is a low-risk or high-risk skeletal site [264, 265, 269, 270]. A recent systematic review and meta-analysis on return to sport following low-risk and high-risk BSI of the lower extremity and pelvis found that treatment complication rate was highest in BSI of the femoral neck, tarsal navicular, anterior tibial shaft and fifth metatarsal (considered high-risk anatomic sites) and lowest in the fibula, pubic bone and posteromedial tibial shaft (considered lower-risk sites) [263]. Clinicians should therefore also include the knowledge of whether the BSI is at an anatomic site that is high-risk or low risk in their return to play recommendations [263]. Collegiate athletes with Triad risk factors, including FHOA and low BMD with elevated CRA scores, have higher grade BSIs on MRI and a delayed return to play [189, 271]. Because an athlete who sustains a BSI loses significant time for their sports participation, an athlete with moderate- or high-risk may be motivated to engage in active management of the Triad to facilitate continued successful participation in her sport.

***Evidence-Based Statement:*** BSIs presenting at high-risk sites, including the femoral neck, tarsal navicular, anterior tibial shaft, and fifth metatarsal, have high complication rates and are associated with delayed return to play. ***Grade:*** Level B.

### What is the Role of the Team Physician in the Return to Play Decision for Triad Athletes?

The team physician’s role with Triad athletes is to establish a return to play process, evaluate the athlete’s medical conditions, work with the sports medicine staff to treat and rehabilitate the athlete and return the athlete to play after she is deemed safe. The CRA tool is well suited for use by a physician (Figs. 4, 5) as it incorporates evidence-based risk factors for the Triad [32, 229, 272] and considers the magnitude (or severity) of risk, assigning a point value for risk factors in each Triad spectrum based on risk severity (low, moderate, and high risk). This CRA is then translated into clearance and return to play guidelines for the Triad based on the athlete’s cumulative risk score. Written contracts/Medical Expectations Agreements (Athlete Contracts) are recommended for high-risk athlete management to optimize clarity and understanding of the athlete plan and to hold the athlete accountable [4].

### What is the Relationship Between Injury and the Triad in Adolescent Athletes, and how are these Factors Considered for a CRA Tool for the Adolescent Athlete?

Adolescents may also be at increased risk for BSI at certain stages when growth is accelerated, especially in the presence of additional Triad-related risk factors [273]. Musculoskeletal injuries frequently occur in adolescents, with a prospective injury incidence ranging from 40 to 50% [255, 274–276]. Several risk factors characteristic of the adolescence period of development may promote an increased injury risk. During peak linear growth, the elongation of bone precedes the development of associated connective tissue and muscle mass, which may lead to loss of flexibility, muscle imbalance, and reduced strength. Additionally, due to the fact that bone mineralization occurs after linear growth, bone may become more porous, weaken, and become more vulnerable to fracture, which is consistent with higher rates of fracture during and in the year following peak height velocity [273, 277].

While a dietary regimen that provides sufficient calories and nutrients supports adolescents’ health and development, factors consistent with energy deficiency exhibit associations with increased risk of musculoskeletal injury or BSI. In a study evaluating 331 female high school athletes on 33 interscholastic teams, 43.1% reported a lower extremity musculoskeletal injury during the sports season. Athletes that met criteria for DE were twice as likely to incur an injury as athletes without DE [255]. Among 163 female high school athletes representing eight sports, 37.4% incurred a musculoskeletal injury during the sports season. DE, menstrual irregularity, and low BMD (*Z* score < − 2.0) were independent predictors of injury in the multivariate model [278]. In a prospective study among female adolescent distance runners, 42.7% sustained a lower extremity musculoskeletal injury during the season. Menstrual irregularity and low BMD were significantly associated with increased injury incidence [243]. These findings support clear and consistent associations between Triad-related factors and increased risk of injury to both soft tissue and bone in adolescent athletes and the importance of prevention and management of Triad symptoms.

Sports-specific factors also relate to the Triad and injury risk in adolescent athletes, with leanness sports exhibiting a higher incidence of factors consistent with energy deficiency, low BMD, and BSI. Conversely, research among adolescent endurance runners supports the relationship between prior participation in ball sports, including basketball and soccer, and lower BSI risk [272]. These ball sports involve multi-directional movements with higher impact loads, as compared to endurance running, which provide a stronger osteogenic stimulus to bone, and thus may provide a protective effect. Recent research also suggests the potential consequences of sports specialization and association with increased injury risk in adolescent athletes [275, 279, 280].

In summary, there is evidence that adolescent athletes in high-risk sports are at greater risk for BSI, especially during periods of rapid growth due to a lag in bone mineralization [273]. A multidisciplinary approach to clearance and return to play should include preventive measures such as optimizing bone health in the pediatric/adolescent age group through participation in sports with multidirectional loading, optimizing energy status, following guidelines for consumption of bone building nutrients, and avoiding overtraining. Screening and risk assessment in the adolescent athlete are advisable for clearance and return to play. In addition, enhancing coaching awareness of the Triad and athlete and parent education and knowledge can help optimize bone health and minimize injury. Herein we introduce a CRA tool for implementation in adolescent populations up to 18 years.

***Evidence-Based Statement:*** Adolescents in high-risk sports may be at greater risk for BSI, especially during periods of rapid growth. ***Grade:*** Level B.

### What are the Important Considerations for the Resumption of Activity for Adolescents in Treatment for a Clinical Eating Disorder?

#### General Comments

To date, published guidance on return to play among young athletes has largely been driven by injury or concussion metrics [281], with less emphasis on advisement for return to play in the context of EDs. Although the peak onset of eating pathology occurs during adolescence and emerging adulthood [282, 283], there is little consensus on how to best navigate return to play and ED treatment during this developmental window. Further, for youth with EDs, exercise can present a clinical conundrum. Exercise generally provides benefit for physical and mental health, and the developing adolescent brain [284, 285], but for some, exercise might have been used to initiate weight loss [10, 286], and aspects of the activity itself [287, 288] or the athletic environment [289–291] may have contributed to the origin and/or maintenance of body image concerns and the ED.

In the acute phase of ED recovery (particularly for those diagnosed with EDs associated with low weight), a majority of patients are advised to cease all activity in order to both support renourishment and prevent negative health outcomes (e.g., cardiac event) [292]. However, once weight status and vital signs have stabilized, comprehensive recovery from the ED will necessarily include establishing an adaptive relationship with physical activity [293, 294]. Resumption of normative levels of activity will be important for full and sustained lifelong mental and physical health [285, 295], with even greater urgency typically placed on a return to play for young athletes who are pursuing an athletic career. However, some clinicians, coaches, and others who work closely with adolescents may feel unprepared to navigate return to play for a variety of reasons, including not feeling adequately trained in understanding EDs, or knowing how to best manage them and/or exercise prescriptions [291, 296]. In addition to limited guidance to date for return to play in the context of eating pathology [125, 294, 297], even less guidance has been specifically oriented toward developmental concerns. With the following text as a guide for informing return to play for adolescents with EDs, these recommendations are meant to serve as a template and should be considered only a part of a comprehensive treatment plan. Each young person is unique in their recovery, and any advice should be personalized and taken under advisement with an expert treatment team.

#### What are the Recommendations for Managing Communication?

The primary team navigating decisions around return to play for young patients with EDs should comprise, at a minimum (1) a mental health provider (e.g., clinical psychologist, licensed clinical social worker), who provides evidence-based psychotherapy; and (2) a family medicine physician, pediatrician or adolescent medicine physician who can advise and monitor medical stability and physical readiness. Optimally, this team would also include a nutritional expert and some, if not all, team members would have experience working with athletes. Parents, coaches, and other training staff should be involved and consulted as indicated, relative to age and circumstances [125, 297], and if possible, they should be educated on the mental health aspects of EDs [289, 291].

Strategies for ensuring that pathways of communication remain open can include the use of shared medical records and/or scheduling regular (e.g., monthly) multidisciplinary meetings. While the benefits of a multidisciplinary team cannot be overstated, one team member should be designated to make final decisions based on team consensus. The designee should typically be the mental health provider, based on their expert knowledge of the ED and ability to confirm and monitor psychological readiness to engage in activity.

A unified stance across members of the care team is particularly important under several circumstances. For one, understandable pressures from within the athletic environment (e.g., an upcoming championship game) may present difficult decision points in which even minimal medical risk must be weighed against several factors including, but not limited to, psychological readiness secondary to the ED; physical readiness due to deconditioning following a necessary period of rest; disappointment on behalf of the patient and family; and career advancement for a talented athlete. Another example of where a unified team stance is necessary is when an athlete might need to restore to a higher weight than they have ever been in order to both fully recover from their ED [117, 298] and establish normative endocrine function and menses [299]. Lack of knowledge in treating EDs and/or lack of experience in working with athlete populations who have weight or aesthetic requirements (e.g., wrestling; skating) can contribute to splitting within the provider team. Specifically, a patient or family member might expect that they will return to their premorbid weight during treatment and be reluctant to agree to recommended higher weight estimates. For many adolescents, early specialization in a sport where a lower-weight body ideal is prioritized (e.g., ballet) may have contributed to “falling off” their growth curve even prior to the ED onset; this general weight suppression (secondary to sport) might lead the child and family to expect the estimated weight during ED treatment to be lower than is needed medically. Further, weight estimations in youth with EDs need to account for normative weight gain that should be developmentally expected during adolescence. In these instances, it is important that all treatment team members provide education and consistent rationale for higher weight estimations that are most likely to support adolescent development, ED recovery and prevent relapse [300]. Finally, in communication with the patient and family, the plan for return to play should be both evidence-based but also personalized and culturally informed. To maximize the success of the care plan, recommendations should be provided with cultural humility [301], and honor patients and their families as valuable informants and guides in clinical decision-making [302].

#### What are the Recommendations for Timing of Return to Play Activity?

The timing at which one is ready to return to activity within the context of ED treatment is variable, and based on clinical judgment that accounts for both physical indices (e.g., weight status) and also psychological readiness [125]. Here, we will discuss each in turn and highlight where they intersect. As noted in Sect. 3.5, FBT is the first-line evidence-based outpatient treatment for youth with EDs, followed by CBT-E when FBT is not indicated (e.g., when families are not able to engage in treatment) [298, 303]. Both are considered behavioral treatments whereby the approach is symptom-focused, with a necessary focus on regular eating and weight stabilization. Despite this pragmatic ethos, it is notable that neither of these treatments provides explicit guidance on when and how activity should be re-introduced. In part, this lack of explicit guidance results from the nature of these manualized treatments, such that both were intentionally designed to be used with fidelity, but also tailored flexibly relative to a given patient and case formulation [304, 305].

#### Physical Status Considerations

In FBT, early weight gain during treatment (i.e., 2–2.5 kg in the first month) is a robust and consistent predictor of positive prognosis [306] and across ED treatment samples, higher body weight is an important indicator of sustained recovery over decades [300]. Within this context, return to play presents a paradox, such that increased activity places the patient at risk for a potential caloric deficit with resulting energy deficiency and menstrual dysfunction, but may also *enhance* weight status and musculoskeletal health secondary to muscle-building. Notably, recent genetic work suggests that anorexia nervosa is in fact a “metabo-psychiatric disorder” due to a suggested shared polygenic risk for both the psychiatric illness, but also for elevated metabolism and activity levels [307]. Accordingly, although dietary restriction will contribute to slower metabolism in the general population [308], patients with restrictive EDs experience hypermetabolism when refeeding [309], which is often exacerbated by increased physical activity levels in the context of the illness [310]. For this reason, adjusting activity levels during return to play may also require a notably higher caloric adjustment, both due to residual features of the ED, but also due to normative higher caloric needs during adolescence, particularly among young athletes [311].

A recent systematic review of muscle health in anorexia nervosa showed that even those who were fully weight-restored still exhibit roughly 9% lower muscle mass compared to controls [312]. Moreover, functional consequences of reduced muscle mass appear to persist, even nearly three decades following recovery from the ED [313]. One study of supervised exercise during ED treatment found that targeted strength training increased not only strength but also BMI [314]. Taken together, although exercise interventions in ED treatment are nascent, preliminary data suggest that some benefit in musculoskeletal health may be achieved from a *guided* return to strength training, with no decrement to weight status.

#### Psychological Status Considerations

More generally, among youth, exercise can provide social structure, self-esteem enhancement, and identity development [315]—and among patients with ED, exercise might also provide motivation for recovery. Wielding exercise as a motivator for ED recovery is a nuanced opportunity that should be carefully considered for each patient individually. This situation has potential to be a proverbial “double-edged sword,” such that patients often report more motivation for weight gain only if it is in the service of muscle enhancement*.* Thus, if the motivation to exercise supports recovery (i.e., weight gain) but is still strongly tied to body image concerns (i.e., alleviating dislike of fat), many patients would not be considered psychologically ready to engage in exercise. Paradoxically, some patients will initially not want to engage in any forms of activity that are not intended to enhance muscle or burn calories (e.g., a slow nature walk), a situation which should be openly discussed with the patient and their family. Alternatively, exercise can be employed as a reward, for exampole, return to play will return the adolescent to their team and social network, which can be used to reinforce recovery-oriented behavior. For example, the child might be allowed to attend practice to watch the other players if they eat full meals and snacks*.* Here, the use of exercise reinforcement is largely in the service of recovery and motivated by the promise of social engagement rather than the ED. It is important for the care team to maintain leverage over requiring continued progress toward recovery-oriented behavior, specifically in not allowing an individual to engage in exercise before they have met certain, expected criteria. For example, the patient might be able to return to practice or rehearsal but should not be allowed to participate in competition until weight status is achieved and maintained (as per what is agreed upon in a collaborative return to play contract, described in Sect. 6.8).

### What are the Practical Considerations for Return to Play in Adolescents with EDs?

Although the use of behavioral contracts may have questionable value for a given patient, they can provide particular utility in coordinating expectations across a care team, and in communicating timelines and milestones with a patient and their family. For patients who are navigating return to play, it is essential that the contract requires their willingness to retain progress in eating what is needed to support increased activity (and align with and support their expected weight status). Contracts should also include a timeline for stages of activity that can be resumed (e.g., training vs. competition), with specific contingencies should the patient fall short of certain milestones. In the case that guidelines are needed to detail contingencies, the use of red/yellow/green levels can be useful in indicating pivots in what is allowed, as needed. For example, in a situation where the adolescent is allowed to participate in team practice 3 days per week, a yellow level might be described as a degree of initial weight loss that is permitted (e.g., up to 1 kg). Here, the corrective action should also be specified, for example, reducing activity to two practices per week unless the weight is regained within 2 weeks. Contracts should be detailed, specific, and cover all relevant aspects of return to play including eating behavior and other potential DE behaviors, and the type, amount, duration, and timing of athletic engagement. Ideally, the contract should be initiated by the team lead, and *collaboratively* adapted with other team members, the patient, and their family. In addition, the contract should serve as a guide for discussion during ongoing meetings to assess progress and updated as needed over time.

When implementing any plan of care, there will be inevitable missteps, and this applies when navigating return to play. To ensure the consequences of these missteps are minimized, a few guiding principles are generally advisable for most individuals. First, it is important to start any return to play plan at a slow pace, both to minimize medical risk, but also to allow for swift course correction, should there be a need to do so. Here it is sometimes helpful to conceptualize the ED as a “metabolic injury.” Had the adolescent experienced any other kind of injury (e.g., sprained ankle), return to play would be gradual, and the same approach applies to return to play during recovery from an ED. To optimally promote both physical and psychological recovery, practice should precede competition, with a gradual build toward performance situations over time. For a majority of youth, return to play should start first with socially oriented activity (e.g., family bike ride) rather than solo activities or exercise explicitly focused on promoting weight loss, muscle enhancement, or other specific performance objectives. Finally, a full return to play will generally require at least as long a time as the adolescent has been ill. Anticipating this protracted timeline with follow-up meetings at a regular cadence (e.g., at least monthly for the first few months) will promote continued progress and allow for necessary adjustments to activity level and/or support over time.

## Summary and Clinical Recommendations of the 2025 Update on the Female Athlete Triad

While Triad research needs to continue, there is an equal need for Triad education and evidence-based policymaking as it concerns the prevention and treatment of Triad-related health related issues. The Female and Male Athlete Triad Coalition is committed to supporting both research and education and, notably, has recently partnered with the National Federation of High Schools to develop an online educational program for individuals coaching at the high school level to help educate coaches on the Triad in girls/women and in boys/men. Advances in research and clinical practice since the 2014 Female Athlete Consensus Statement [2–4] have continued to build out the evidence-based conceptual model of the Triad, improving its specificity, scalability, and accuracy. This update introduces revised clinical guidelines based on the latest scientific and clinical literature and, notably, introduced a new model and new clinical guidelines focused specifically on the adolescent athlete. Regarding the components of the Triad, the terminology used for energy status has been revised with the introduction of the concept of metabolic compensation, and additional criteria for the determination of energy deficiency have been included. Regarding EDs and DE, the update includes information pertaining to the use of assessments of eating attitudes and behaviors using standardized questionnaires as additional proxies to aid in identifying energy deficiency. Recommendations for screening, diagnosing and treating ED based on the DSM-5 are updated, stressing the importance of a mental health evaluation. With respect to menstrual function, data presented on the magnitude of energy deficiency that is causally related to the induction of menstrual disturbances, the pace of induction of and recovery from menstrual disturbances, and definitions of various stages of menstrual recovery all send a message to clinicians that the resumption of menses alone does not indicate full recovery of ovulation and ovarian steroid exposure. The importance of identifying women with high FAI is emphasized, and the potential for gynecological age and psychological stress susceptibility to modify the risk of the Triad is discussed. Bone health updates include recommendations for sites to be DXA scanned, definitions of low BMD, prognostic factors to consider with BSI evaluation (i.e., MRI grading, bone type, and skeletal site), and new information on hormone replacement therapy and other pharmacological strategies to treat low BMD. Lastly, the CRA tool has been updated for use with clearance and return to play decision-making for adult athletes and a new tool for use with adolescents has been introduced. All evidence-based statements are compiled in Table 12.

**Table 12:** Clinical guidelines for screening, diagnosis, treatment and return to play for adolescents and adults: evidence-based statements

Current recommendations to screen for and diagnose energy deficiency
**Evidence-Based Statement:** Measures of energy deficiency are most useful when obtained serially within an individual at the same time of day
***Evidence grade:**** Level B* **Evidence-Based Statement:** Evidence of energy deficiency is best obtained by documenting serial decreases in at least one or more of the following physiological or behavioral measures: body weight, % expected body weight, BMI, percentile BMI for age (adolescents), EA, or increases in measures of eating attitudes and behaviors reflective of dietary restriction (see Sect. 3) and if resources allow, measures of metabolic compensation such as decreases in the RMR ratio, and or decreases in fasting endocrine measures such as total or free T_3_, IGF-1, leptin, and or increases in ghrelin ***Evidence grade:**** Level B* **Evidence-Based Statement:** If a single time point is all that is available, measurements of body weight or BMI may indicate a high risk of undernutrition or chronic energy deficiency if body weight is < 85% of expected body weight, or BMI is < 17.5 kg/m^2^, EA is much less than 45 kcal∙kg FFM^−1^∙day^−1^, if an adolescent’s BMI for age is < fifth percentile, or if the RMR ratio or TT_3_ are low ***Evidence grade:**** Level B*

Assessment of eating attitudes and behaviors as a proxy indicator of energy status
**Evidence-Based Statement:** Psychometric measures of eating attitudes and behaviors, e.g., drive for thinness and/or dietary restraint, can serve as useful proxy indicators of energy deficiency
***Evidence grade:**** Level B* **Evidence-Based Statement:** Behaviors such as compulsive exercise, inadvertent undereating, time restricted eating e.g., intermittent fasting and/or undereating for prolonged periods during the day are associated with energy deficiency ***Evidence grade:**** Level C*

Screening for Triad risk factors and ED/DE
**Evidence-Based Statement:** All female athletes should be screened for Triad risk factors. The Preparticipation Physical Evaluation (PPE), administered by health care providers, can effectively assess bone health, menstrual dysfunction, and ED/DE risk; other psychometric tools should be considered to assess ED/DE attitudes and behaviors, serving as a proxy for energy status
***Evidence Grade:**** Level B* **Evidence-Based Statement**: When an ED or DE is suspected, best practices should include evaluation by providers from three specialty areas: physician (medical concerns), registered dietitian, preferably a sports dietitian (nutritional concerns), and mental health professional, preferably a provider with ED expertise, (mental health concerns) ***Evidence Grade:**** Level B* **Evidence-Based Statement:** Individuals with OSFED, including atypical anorexia nervosa, may not present at a low weight, relative to population-based norms, but may experience very serious medical and psychological consequences secondary to malnutrition ***Evidence Grade:**** Level A* **Evidence-Based Statement:** Athletes can be diagnosed with all types of ED, present with any weight, and exhibit any of the behavioral features of recognized ED diagnoses ***Evidence Grade:**** Level A*

Treatment for ED/DE
**Evidence-Based Statement:** Treatment for ED among athletes should be designed to minimize acknowledged barriers to entry and address athlete and sport-specific factors that contribute to engagement in DE, and treatment responses
***Evidence Grade:**** Level B* **Evidence-Based Statement:** Current psychotherapy treatments for athletes remain largely untested, but evidence suggests that standard ED care is also appropriate for athletes ***Evidence Grade:**** Level B*

Diagnostic and treatment updates to the reproductive function continuum
**Evidence-Based Statement***:* In some exercising women with menstrual disturbances, energy deficiency may co-occur with hyperandrogenemia
***Evidence Grade:**** Level B* **Evidence-Based Statement:** Hypothalamic inhibition of the hypothalamic pituitary ovarian axis secondary to energy deficiency may restrain the typical symptoms of hyperandrogenism in exercising women ***Evidence Grade:**** Level B* **Evidence-Based Statement:** Clinicians should consider a more comprehensive approach to defining endpoints for reproductive recovery in response to a nutritional intervention including the goal of achieving successive menstrual cycles with lengths of < 36 days and increasing ovarian steroid concentrations ***Evidence Grade:**** Level A* **Evidence-Based Statement:** A nutritional intervention designed to increase energy intake between 300–600 kcal/day will likely *initiate* reproductive recovery. However, factors such as menstrual history, baseline menstrual status, i.e., amenorrhea or oligomenorrhea, baseline body weight, the possibility of relapse, and lifestyle factors such as psychosocial stress are predictors of the timing and success of such interventions ***Evidence Grade:**** Level B*

Diagnostic and treatment updates to the bone health continuum
**Evidence-Based Statement:** We recommend assessing BMD with a DXA scan based on details in Table 7
***Evidence Grade:**** Level A* **Evidence-Based Statement:** We recommend optimizing weight gain and resumption of menses by reversing energy deficiency (by increasing caloric intake, improving nutrition, and/or decreasing exercise energy expenditure) in exercising adolescents and women with energy deficiency, menstrual dysfunction, and low BMD associated with the Triad ***Evidence Grade:**** Level B* **Evidence-Based Statement:** We recommend a multidisciplinary approach to support the athlete/exerciser and achieve nutritional goals; this should include at least a primary care and/or sports medicine physician, and a registered dietitian, preferably a sports dietitian. Additional consultation may be obtained from a mental health practitioner, an endocrinologist, an orthopedic surgeon, a psychiatrist, an exercise physiologist, a certified athletic trainer, family members and/or the team coach ***Evidence Grade:**** Level A* **Evidence-Based Statement:** We recommend optimizing calcium and vitamin D status ***Evidence Grade:**** Level B* **Evidence-Based Statement:** We recommend continuing non-pharmacologic therapy for at least 6–12 months before considering other therapies unless bone health deteriorates over this period or new fractures arise ***Evidence Grade:**** Level C*

Cumulative risk assessment for clearance and return to play: assessment tool and guidelines
**Evidence-Based Statement:** There is a dose–response relationship with Female Athlete Triad risk factors and bone in that the higher the number of cumulative risk factors, the greater risk for low BMD and/or BSI risk
***Evidence Grade:**** Level B* **Evidence-Based Statement:** The Triad Coalition’s CRA score is predictive for BSI in female athletes ***Evidence Grade:**** Level B* **Evidence-Based Statement**: It is proposed that women who present with objective physiological criteria of metabolic compensation consistent with prolonged energy deficiency be categorized, at minimum, as “Provisionally Cleared” ***Evidence Grade:**** Level C* **Evidence-Based Statement:** Female athletes in lean sports have a higher prevalence of Triad risk factors than those in non-lean sports ***Evidence Grade:**** Level B* **Evidence-Based Statement:** BSIs of higher MRI grades are associated with a delay in return to play compared to BSIs of lower MRI grades ***Evidence Grade:**** Level B* **Evidence-Based Statement:**The Triad CRA tool score is positively associated with the risk of BSI of trabecular-rich skeletal sites ***Evidence Grade:**** Level B* **Evidence-Based Statement:** Nutrition intervention programs may reduce BSI, especially in trabecular-rich skeletal sites ***Evidence Grade:**** Level B* **Evidence-Based Statement:** BSIs presenting at high risk sites, including the femoral neck, tarsal navicular, anterior tibial shaft, and fifth metatarsal have high complication rates and are associated with delayed return to play ***Evidence Grade:**** Level B* **Evidence-Based Statement:** Adolescents in high-risk sports may be at greater risk for BSI, especially during periods of rapid growth ***Evidence Grade:**** Level B*

*BMI* body mass index, *EA* energy availability, *ED/DE* eating disorders/disordered eating, *RMR* resting metabolic rate, *T*_*3*_ triiodothyronine, *IGF-1* insulin-like growth factor-1, *kg* kilogram, *FFM* fat-free mass, *OSFED* other specified feeding or eating disorder, *BMD* bone mineral density, *DXA* dual-energy X-ray absorptiometry, *BSI* bone stress injury, *CRA* clinical risk assessment tool, *MRI* magnetic resonance imaging

Building on the Evidence-Based Statements Presented in the Updated 2025 Consensus Statement, the Following Summarizes the Recommendations:

- Exercising girls and women should be educated about the health problems that can result from failing to fuel their bodies adequately to meet their energetic requirements for total energy expenditure.
- Assessments of energy deficiency are critical to identifying Triad risk and use of the revised terminology to categorize the severity/risk of energy deficiency is recommended.
- Sports medicine clinicians should be aware that the simple return of menses does not indicate that regular ovulation has resumed, or that ovarian steroid concentrations have normalized. Several consistent cycles of regular length are more indicative of full menstrual recovery.
- Exercising girls and women should be screened and monitored for risk factors and at-risk athletes should be identified and referred to a sports dietician, physician, and/or eating behavior and possibly a mental health practitioner to prevent exacerbation of at-risk behaviors.
- Sports medicine clinicians and practitioners should be specifically trained and educated on up-to-date Triad science and adopt evidence-based approaches to Triad prevention and treatment.
- Translational tools such as the PPE, validated eating behavior questionnaires, the Triad clearance and return to play algorithm, and if resources allow, laboratory measurements of energy deficiency, should be incorporated into sports medicine teams’ approaches to Triad prevention and management.
- Female athletes with moderate to high Triad risk should receive coordinated care from an experienced multidisciplinary treatment team consisting of a physician, registered dietitian, and mental health practitioner (especially if the athlete has suspected disordered eating, eating disorder).
- Sports medicine clinicians should utilize MRI grading for assessment of BSI risk (low MRI grade vs. high MRI grade) and for guidance in return to play decisions.
- The clinician should be knowledgeable of high risk versus low-risk skeletal sites and take this into consideration in their return to play decision making.
- Nutrition intervention programs are recommended for reducing risk for BSI, especially in trabecular-rich skeletal sites.
- Triad risk assessment tools are recommended for the female adolescent athlete for clearance at the pre-participation physical and for return to play decision making.

## Conclusion

The 2025 Update to the Female Athlete Triad Coalition Consensus Statement Part 2 provides the most current and evidence-based information on the prevention, treatment, and return to play decision making pertaining to girls and women with Triad conditions. These recommendations reflect advances in Triad science and supersede the previous 2014 Triad Consensus Statement [4]. Clinicians, researchers, and sports medicine practitioners are also referred to the 2025 Update to the Female Athlete Triad Coalition Consensus Statement Part 1 [1] companion paper for a more in-depth review of the scientific basis of these clinical guidelines.

## Declarations

### Disclaimer

The opinions and assertions expressed herein are those of the author(s) and do not reflect the official policy or position of the Uniformed Services University or the Department of Defense. The contents of this publication are the sole responsibility of the author(s) and do not necessarily reflect the views, opinions or policies of The Henry M. Jackson Foundation for the Advancement of Military Medicine, Inc. Mention of trade names, commercial products, or organizations does not imply endorsement by the U.S. Government.
